# PG-18: turtles reach adult shell shapes at about 65% maximum carapace length

**DOI:** 10.1186/s13358-025-00395-0

**Published:** 2025-08-05

**Authors:** Guilherme Hermanson, Serjoscha W. Evers

**Affiliations:** Department of Geosciences, University of Fribourg, Fribourg, Switzerland

**Keywords:** Ontogeny, Shell shape, Shape change, Turtles, Morphometrics, Allometry, Sexual dimorphism

## Abstract

Ontogenetic shell shape changes of turtles are often only documented for individual species. It is currently unclear how shell shape changes during ontogeny across species, if there are common trends, and at what point in ontogeny individuals reach their adult morphology. Inspired by questions of whether some morphologies are too juvenile to be included into macroevolutionary studies of shell shape, we develop ontogenetic shell shape curves based on landmarked 3D shell shapes of turtles. Species-specific allometric shape regressions confirm that turtles show marked ontogenetic shell shape change. Geometric morphometric analysis shows that juvenile turtles have rounded shells, and ontogenetic differentiation between species increases adult turtle disparity. Disparity analysis indicates that juvenile shells across turtle clades are more similar than adult shapes, suggesting an important role of developmental constraints on early turtle shell shape, and possible adaptive post-natal ontogenetic changes that produce the observed adult shell shape disparity. Ontogenetic shell shape curves indicate when turtles converge onto adult morphologies, here quantified as 85% the distance between juvenile shape and maximum size adult shape. This happens at about 65% of the species-specific maximum carapace sizes. Sexual shell shape dimorphism is comparatively low across turtles even in the presence of pronounced sexual size dimorphism. These preliminary results provide guidance for studying shell shape macroevolution, but need to be scrutinized further in the future by data addition.

**Supplementary Information:**

The online version contains supplementary material available at 10.1186/s13358-025-00395-0.

## Introduction

Many aspects of morphology are known to vary throughout ontogeny. This is why comparative studies ideally use adult morphologies, or individuals from the same size class. For turtles, skull shape (e.g., Bever, 2009; Chatterji et al., 2022; Mariani & Romano, 2017), skull topology (Miller et al., 2024), limb shape (Hermanson et al., 2024; Stevens et al., 2018), shell ossification (Joyce, 2025; Pritchard, 2008), bone surface sculpturing (Joyce, 2025; Joyce et al., 2018; Massonne et al., 2023), scute patterns (Cadena et al., 2021), shell shape (e.g., Angielczyk & Feldman, 2013; Burroughs et al., 2013; Cadena et al., 2021; Cordero et al., 2018, 2019; Fish & Stayton, 2014; Gardner & Russell, 1994; Kordikova, 2002; Pate & Salmon, 2017; Stevens et al., 2018) amongst other traits are known to change ontogenetically to various degrees. In morphological phylogenetic studies, scoring of characters is often explicitly recommended for adult morphologies (e.g., Joyce, 2025; Meylan, 1987). Ontogenetic changes also pose a potential problem for comparative shape analyses, especially when shape changes are associated with further life-history changes, such as dietary niche shifts (e.g., Chatterji et al., 2022; Jones & Seminoff, 2013; Nishizawa et al., 2010; Pate & Salmon, 2017). However, samples are often not ontogenetically standardized prior to analyzing aspects of interspecific shape variation, leading to pervasive datasets that include juveniles and adults (Dziomber et al., 2020; Hermanson et al., 2022; Stayton, 2024; Stayton et al., 2018). In this case, the (often untested) assumption is that interspecific variation is larger than intraspecific shape differences associated with ontogeny (Foth et al., 2017; Hermanson et al., 2022; Nishizawa et al., 2010). Other studies have established size thresholds and exclude ‘juvenile’ data for example based on size arguments (e.g., Hermanson & Evers, 2024), osteological features (Hermanson et al., 2024) or morphofunctional arguments (Stayton et al., 2018), leading to non-standard classifications of ‘adult’ morphologies across studies. However, shape changes in the shell have not been documented so far for a larger number of turtle species, making it difficult to establish if it is possible to infer a single size threshold upon which a turtle shell can be labelled as representing the ‘adult’ morphology.

Being faced with this question for a different study, we exploited ideas to find a more rigorous way of assessing adult shell shape morphologies. Using a previously published shell shape dataset that includes broad size ranges (Stayton, 2024), we develop a preliminary method that compares shell shapes across shell sizes taken as ontogenetic proxies. We find that all tested turtle species show shape changes in their shell, but that shell shapes converge toward a common shape at about 65% of the maximum recorded shell length. Although there are exceptions to this rule, our preliminary data offers a pragmatic solution for size threshold decisions in comparative studies on turtle shells, and provides many avenues for future refinement.

## Material and methods

### Institutional abbreviations

AMNH, American Museum of Natural History, New York, New York, USA; FLMNH, Florida Museum of Natural History, Gainesville, Florida, USA; FMNH, Field Museum of Natural History, Chicago, Illinois, USA; KU, University of Kansas Biodiversity Institute, Lawrence, USA; USNM, National Museum of Natural History, Washington, DC, USA.

### Morphometric dataset

We used the 3D landmark dataset of turtle shells from Stayton (2024), which includes 3095 specimens of 306 species as a basis to extract a smaller dataset on which ontogenetic shell shape change can be tested. Although the dataset of Stayton (2024) was not specifically designed for quantifying ontogenetic variation, many species are sampled by multiple juveniles, subadults, and adults. Thus, we think that the dataset is a good starting point to explore ontogenetic shape variation in a preliminary assessment. To test if species exhibit significant shell shape changes throughout ontogeny, we first subset the dataset of Stayton (2024) to only include species for which there were at least 8 specimens and for which there was at least a three-fold difference between the smallest and largest individuals (N = 65 species). From this, we further excluded four species (*Graptemys barbouri*, *Graptemys pulchra*, *Hardella thurii, Elseya irwini*) in which the size range was effectively only so large because males and females have extremely large sexual size dimorphism. The remaining dataset includes 61 species and 913 specimens.

To increase the sampled size ranges for several of these species, we landmarked an additional 79 specimens using the same landmarking concept of Stayton (2024; Supplementary Fig. 1). This was done in the Avizo software (v. 9, Visualization Sciences Group). The new 3D coordinates were then imported to R (R Core Team, 2021) using custom code (Hermanson, 2025) and bound to the original 3D landmark dataset. We also included the shell length for one specimen (*Trachemys gaigeae* KU 51203) because it had no data in the original dataset. This value was calculated as the distance between the first and twelfth landmarks, which describe the anterior and posteriormost points of shells in the original landmarking concept. For two species, we excluded the largest specimens because they were larger than the maximum recorded shell lengths for these species (*Cyclemys oldhamii* USNM 328006*; Podonemis vogli* FMNH 73416; TTWG, 2021), making it possible that these specimens are misidentified.

The final dataset was subjected to a Generalized Procrustes Analysis (GPA; Gower, 1975) with the gpagen() function in geomorph (Baken et al., 2021) to obtain Procrustes-aligned 3D data free of translation, orientation and size effects intrinsic to the original data. We then performed a Principal Component Analysis (PCA) to ordinate the final 3D-aligned data in major axes of shape variation (principal components; PCs) which were used in downstream analyses (see below ‘Disparity analysis’).

### Allometric regressions

To test the effect of body size on shape, we performed allometric regressions, using the procD.lm() function in geomorph (Baken et al., 2021). The log_10_-transformed centroid size output from the GPA step was used as the size proxy for this analysis. For 21 species in which sexes were fairly evenly distributed along the size variation for the species, we repeated the allometric regressions but included sex as a covariate. This was done to test the relative effects of ontogenetic shell shape variation and sexual shape dimorphism.

### Shell shape proxies

We extracted the common allometric component (CAC; Mitteroecker et al., 2004) values from our species-specific pure-allometric procD.lm models as one of our two shell shape proxies for downstream applications using the plotAllometry() function (Baken et al., 2021). CAC values represent regression scores that describe shape changes associated with size (e.g., Friedman et al., 2019; Mitteroecker et al., 2004; Orbach et al., 2018). As an alternative to assessing ontogenetic shape change, we also use a multivariate way of extracting allometric shape trajectories for each species from our linear models, using the PredLine method of the plotAllometry() function (Baken et al., 2021). This provides a shape trajectory along size based on the available shape-size data in each species-specific model. For downstream usage, we abbreviate this as ‘multivariate shape’.

### Visualization of ontogenetic shell shape change

For graphical examination of shape variation between small and large-sized specimens for each species we used the shape.predictor() function in geomorph (Baken et al., 2021) and used the CAC values as linear predictors. This produced landmark configurations of shell shapes associated with 5% and 95% percentiles of CAC values as proxies for our ‘small-sized’ and ‘large-sized’ specimens (landmark configurations were identical when using multivariate shape values). Lastly, we also computed normalized Euclidean distances (i.e., 0–1 range) between these two landmark configurations to visually assess the magnitude of morphological differences between individual points, whereby low values (closer to 0) indicate few modifications and large values (closer to 1) represent greater variation.

### Ontogenetic shell shape curves

We wanted to visualize how shell shape changes during ontogenetic growth. Although we use centroid size for allometric regressions, we use straight carapace length as our ontogenetic proxy in our ontogenetic shell shape graphs. SCL is intrinsically available for all of our data, directly correlates with centroid size, but is intuitively easier to understand and is also an observable biological property of each specimen.

To test if specific shapes represent adult morphologies, we require a definition of ‘adult’, which is maybe best measured in terms of size. However, it is difficult to know at what carapace length species can be considered fully grown (i.e., ‘adult’), because reptiles continue to grow throughout their lifetime (e.g., Congdon et al., 2013), although growth becomes arrested at some point (e.g., Andrews, 1982; Frýdlová et al., 2020; Schoener & Schoener, 1978). The TTWG (2021) compiled a list of maximum recorded straight carapace length for all species. A record-sized turtle can be safely assumed to be adult, but record maximum shell size values per definition exceed common adult body sizes (e.g., Moll, 1986; Souza & Abe, 2001), although it is rarely documented by how much. Thus, maximum straight carapace length would be a very conservative threshold to determine adulthood. Here, we commonly use and report specimen sizes as the percentage proportion of the maximum-recorded SCL size of that species, recognizing that even values that fall far short of 100% may represent adults. Sexual maturity in turtles is often reached at around 60% of the maximum straight carapace length (Avens & Snover, 2013; Hermanson & Evers, 2024; Kuchling, 1988; Mosimann & Bider, 1960; Webb, 1956). Thus, as an initial guide, it may be reasonable to assume that SCL ranges between 60 and 100% of the maximum reported SCL document adult size ranges. However, shell shape may well change after sexual maturity of a turtle is reached.

In our ontogenetic shell shape graphs, we used CAC values as well as multivariate shape as our shape proxies. The graphs were produced as biplots of CAC values or multivariate shape scores, plotted against straight carapace length (SCL). The relationships of these plots are conceptually similar to growth curves, in which we expect rapid growth followed by a stationary phase in which shape scores show a horizontal plateauing after a certain size threshold is reached (e.g., Cordero, 2023; Lindeman, 1999). Only if shape change continues to accumulate with growth throughout all life, we expect a linear relationship of shape values and size increase up into the largest size classes of species. It is the ontogenetic size threshold at which shape values start to converge that we wanted to extract from the data.

To visualize the ontogenetic shell shape trends, we fit Gompertz functions with the drm() function of the drc package (Ritz et al., 2015) to each species-specific dataset and shape proxy, which are logistic curves used to describe growth. The fitted curves will flatten if the data reaches a stationary phase. The shape of the Gompertz curves will be influenced by the datapoints available for study, and our data was not evenly distributed across the covered SCL ranges. Thus, due to the overall low and uneven data coverage, the Gompertz curves (and thus the point at which stationarity of shape values is reached) may have a large uncertainty in this preliminary study, depending on the species. However, as the Gompertz curves do not enforce a sigmoidal trend when the data distribution does not reach an asymptote, this can be used to inspect if datasets are either too poorly sampled to infer shape convergence during adult stages, or if shape change does not follow a Gompertz function.

### Minimum size thresholds for adult shell shapes

For each Gompertz function, we assess the asymptotic values, extracted with the coef() function of the stats package (R Core Team, 2021). We then computed 90% confidence intervals around the asymptotic value and show the lower 5% interval as a horizontal line in the ontogenetic shell shape curves. Intersections of the confidence threshold with the Gompertz function indicate the size threshold at which 95% of the stationarity asymptote of shell shape values are reached. For species in which a stationary shape phase was not apparent in our plots, we did not infer a minimum size threshold (i.e., horizontal lines are absent from the respective plots).

In addition, we test a second way of determining the size threshold at which adult shape is reached. For this, we use shape distances between the smallest specimens available to us and predicted shapes of the largest-recorded body sizes of all species. For this, we used the shape.predictor() function of geomorph (Baken et al., 2021) to predict 1000 shapes along 1000 size values ranging from our smallest specimen of each species to the largest ever recorded size for each species, using the CAC or multivariate shape scores. For species with strong sexual size dimorphism, we used the larger sampled sex for predictions. Then we ran a second regression between the results of these predictions and size to extract the allometric trajectory, from which we then determined the size threshold at which 85% of the Procrustes distance between juvenile shape and largest adult shape was reached. As the largest ever recorded specimen sizes are often exceptions in terms of size, we set a threshold of 85%, which is still conservative with regard to the 60% size threshold at which turtles commonly reach sexual maturity. In effect, this means that 15% shape difference to largest possible species size is still accepted as an adult morphology. Possibly, a better approach would be to take a more conservative threshold (of 95%) around the shape means of adults. However, in the absence of average adult size data for most species, modelling a less strict threshold around extreme sizes (which are reported for all turtle species) seem a good compromise for this preliminary study. These thresholds are indicated by vertical red lines in our graphs.

Size thresholds at which adult shapes are reached were calculated in terms of %-size of the maximum recorded carapace lengths for each species and sex (TTWG, 2021), in order to try establishing a minimum threshold representative of a ‘large’ shell morph. Depending on the sample, our minimum thresholds are either for both sexes (when sexual size dimorphism is absent), or for one sex only (when species have pronounced sexual size dimorphism). This is indicated in each graph.

### Cluster analysis

The ontogenetic shell shape plots suggested that many species show ‘stages’ of shape development that correspond to different rates of shell shape change along trends, evident by relatively strong changes of shape scores over a relatively minor carapace-length range. Gompertz curves predict three principal phases of shape development: an early phase with low change rates, followed by a second phase of rapid shape change, followed by a third phase of shape convergence onto an adult morphology. Here, we conceptualize these as ‘small’, ‘intermediate’ and ‘large’ shell morphotypes during ‘juvenile’, ‘subadult’ and ‘adult’ life phases. To see if shape and size data provide evidence for three phases in which the last phase corresponds to a stationary adult phase, we ran a cluster analysis on each dataset and plotted the resulting cluster aggregations in our ontogenetic shell shape curves. This allows us to assess visually, if the ‘large’ cluster falls within the stationary part of the Gompertz curves and/or within the 85% adult-shape range. To perform cluster analysis, we computed pairwise Euclidean distances between CAC values/multivariate shape values and shell size values within each species to obtain a dissimilarity structure (i.e., a list of distances between pairs of specimens). These distances are then repeatedly grouped together based on similar values until within-cluster variances are minimal in the final cluster aggregations generated (Davis, 1986). We defined three clusters a priori that would represent the ‘small’, ‘intermediate’ and ‘large’ stages within each species. This was done using the dist() and hclust() function in stats (R Core Team, 2021). Results of the cluster analysis were used for disparity analysis downstream (see below), and also to inspect if adult shape thresholds and/or shape proxy stationarities are reached already during the ‘intermediate’, or only during the ‘large’ size stages.

### Disparity analysis

Initial shape analyses suggested that juvenile turtles are more similar to one another than adult turtles suggesting shape differences to arise only later during ontogeny. This hypothesis is possibly also supported for turtles by independent studies, for instance with regard to skull shape of juvenile vs. adult sea turtles (e.g., Chatterji et al., 2022). To test this, we applied disparity tests to see if juvenile turtles occupy a smaller shape space than adults. For this, we used the PC scores to calculate the morphological disparity of ‘small’ (N = 195), ‘intermediate’ (N = 367) and ‘large’ (N = 351) clusters of specimens as grouped by the cluster analysis. The cluster sizes show that each group, including the ‘small’ cluster, have adequate sample size for disparity analyses. We defined sum of ranges (SoR) as one of our disparity metrics, because it reflects the total variation observed and because we were interested in the total spread of shape variation exhibited by the different groups (Wills et al., 1994; Wills, 1998; Hopkins & Gerber, 2017). As a second disparity metric, we calculated Procrustes variances on the Procrustes-aligned shell shape coordinates, which correspond to how much specimen shapes differ from their (ontogenetic) group mean shape (Zelditch et al., 2012). SoR was computed using the dispRity package (Guillerme, 2018), and data was both bootstrapped and rarefied as recommended for range-based disparity analyses (Hopkins & Gerber, 2017). Rarefaction is also important because ‘large’ specimens had nearly twice as many specimens compared to the ‘small’ ontogenetic group. Procrustes variances were calculated using the morphol.disparity() function of geomorph (Baken et al., 2021) and also bootstrapped to obtain disparity distributions. We assessed the statistical difference between pairwise group disparities using the overlapping confidence intervals (CI) test of Zou (2007). This approach quantifies how distant midpoints of CIs are from one another, while considering their standard errors, thus providing an estimate of how much overlap there is among the disparity ranges of each group. The resulting p-values for the significance of the overlaps were adjusted using a Bonferroni correction.

## Results

### Turtles show shell shape changes throughout ontogeny

Most turtle species in the analysed dataset exhibit statistically detectable shell shape changes associated with size increase (N = 50 out of 61), as shown by significant results of allometric regressions (p = 0.001–0.046). The strength of these relationships varies between species (R^2^ = 0.09–0.46; Z-score = 1.67–4.09; Supplementary Table 1). All species-specific datasets were relatively small (N = 8–29), so that specific size classes were sometimes only represented by a single specimen. Thus, it is unsurprising that our datasets are sensitive to data inclusion/exclusion as indicated by tests in which we deleted selected datapoints, and additional data inclusion would likely increase the statistical power to detect ontogenetic shape change trends (e.g., McShane et al., 2019). We think that small sample sizes can also explain the non-significant results for eleven out of the 61 species analysed. In four of these, results were marginal to the nominal 0.05 p-value threshold (0.051–0.082). This is supported by visual inspections of shell shapes across size classes for these species, which also showed that they had similar shape change patterns to turtles with significant results (e.g., shell lengthening, changes in vertebral scutes form; Supplementary Figs. 2–12). Adding further data along the size gradient in the future may thus refine our regression results. Nevertheless, our datasets can show that there are significant shell shape changes along ontogenetic growth series.

### Turtle shells become relatively longer and narrower as they grow

**Fig. 1:**
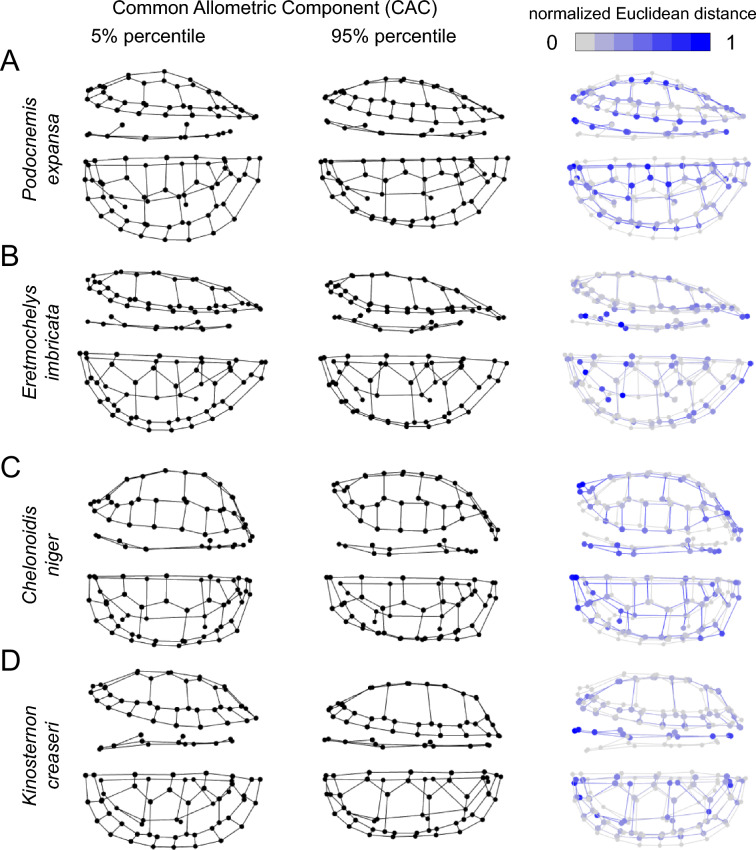
Ontogenetic shape changes in turtle shells across selected species. Landmark configurations shown for the 5% (left column) and 95% (middle column) percentiles of CAC values of **A** *Podocnemis expansa*, **B** *Eretmochelys imbricata*, **C** *Chelonoidis niger*, and **D** *Kinosternon creaseri*. Right column shows normalized Euclidean distances between landmarks of each configuration, whereby bluer points in the colour gradient denote greater morphological change. For each species, shells are shown in left lateral (top rows) and dorsal views (bottom rows)

We observe ontogenetic shell shape changes common to most turtles as well as species-specific changes. The predominant change apparent in most species is a change from a rounded juvenile shape to more elongate and narrow shell shapes in adults (Fig. 1). This indicates negative allometry of shell width, i.e., rates of shell growth are larger for the anteroposterior shell axis than for the transverse axis. Also common to most turtles are ontogenetic changes in the vertebral scute shape at varying degrees, possibly as a consequence of overall shell lengthening.

We do not discuss species-specific changes in detail here. However, the species used for illustration purposes also document changes in other aspects of shell shape. For instance, the highly aquatic *Podocnemis expansa* and *Eretmochelys imbricata* develop relatively lower-domed shells throughout ontogeny (Fig. 1A, B), whereas in larger individuals of *Chelonoidis niger* the nuchal region exhibits a more prominent upward arching and the posterior region of the carapace outer rim becomes more upturned (Fig. 1C). Hinged turtles, such as the kinosternid *Kinosternon crease*ri (Fig. 1D) or *Pelusios* spp. (Supplementary Figs. 5, 9) show particularly strong changes in the anterior plastral lobe.

### Ontogenetic shell shape trends in turtles

**Fig. 2:**
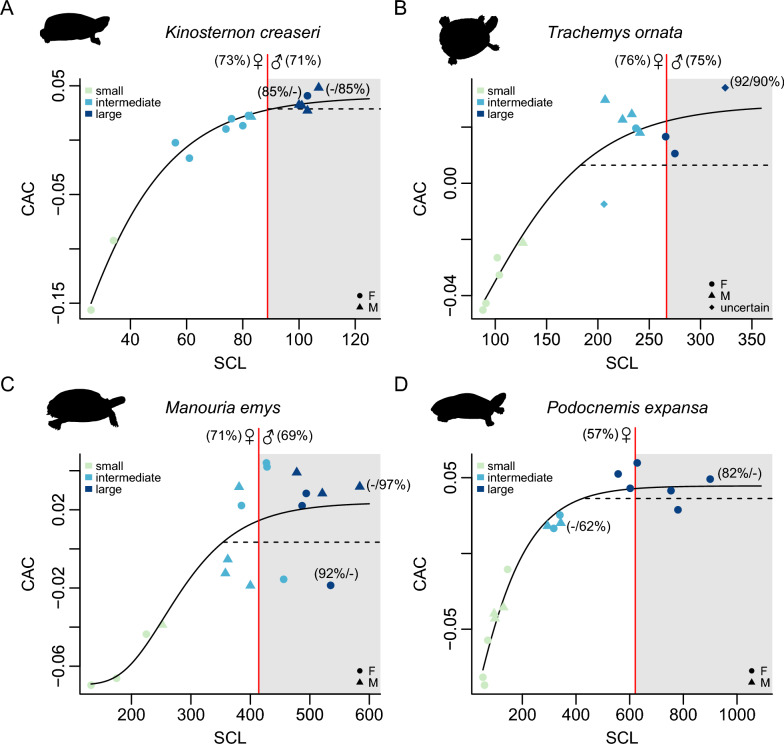
Ontogenetic shell shape curves for selected species. Bivariate plots of the common allometric component (CAC) against straight carapace length (SCL, in mm). **A** *Kinosternon creaseri*, **B** *Trachemys ornata*, **C** *Manouria emys*, **D** *Podocnemis expansa*. The size-range for adult CAC shape, based on our 85%-adult shape threshold, is indicated by grey boxes. The red vertical line indicates the size threshold at which CAC values reach 85% of the shape of record-sized specimens, with corresponding female and male percentages of maximum SCL indicated at the top of the line. Datapoints are colour-coded according to their cluster assignments (‘small’, ‘intermediate’ or ‘large’), and symbols represent sex. (X/Y%) values at largest datapoint indicates percentage of maximum female/male SCL recorded for the species. Solid lines are Gompertz curves fit across all data points. Dashed horizontal lines represent the lower 95% interval at which the asymptotic CAC value is reached according to the Gompertz functions

**Fig. 3:**
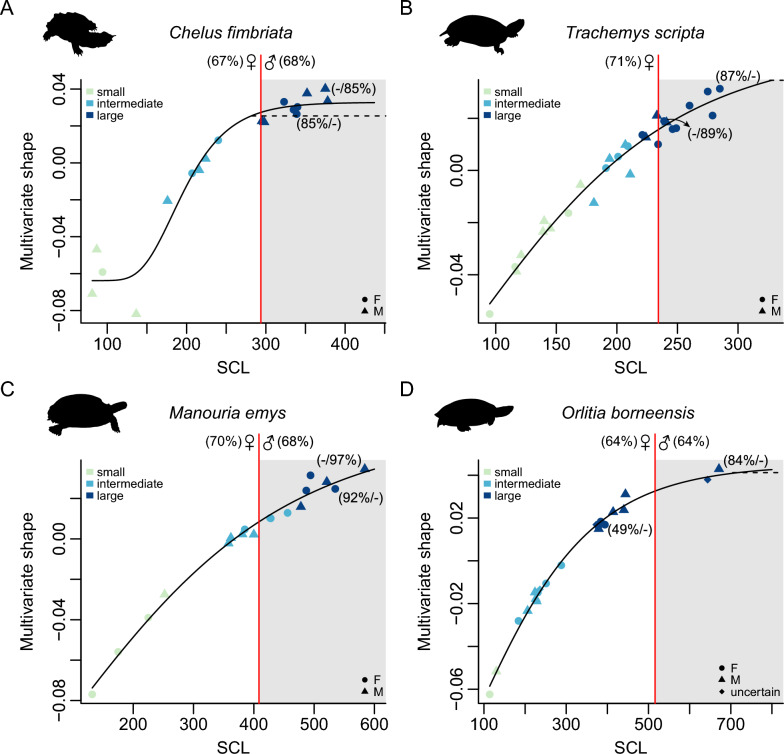
Ontogenetic shell shape curves for selected species. Bivariate plots of multivariate shape against straight carapace length (SCL, in mm). **A** *Chelus fimbriata*, **B** *Trachemys scripta*, **C** *Manouria emys*, **D** *Orlitia borneensis*. The size-range for adult multivariate shape, based on our 85%-adult shape threshold, is indicated by grey boxes. The red vertical line indicates the size threshold at which multivariate shape values reach 85% of the shape of record-sized specimens, with corresponding female and male percentages of maximum SCL indicated at the top of the line. Datapoints are colour-coded according to their cluster assignments (‘small’, ‘intermediate’ or ‘large’), and symbols represent sex. (X/Y%) values at largest datapoint indicates percentage of maximum female/male SCL recorded for the species. Solid lines are Gompertz curves fit across all data points. Dashed horizontal lines represent the lower 95% interval at which the asymptotic multivariate shape value is reached according to the Gompertz functions

Our ontogenetic shell shape curves visually show how shape changes are distributed along size increases (Figs. 2, 3; Supplementary Figs. 13–17).

85% of the maximum adult CAC values are reached at a median size of 67% (min_CAC_ = 57%; max_CAC_ = 75%). This is nearly identical to the median size threshold at which 85% of maximum adult multivariate shape is reached (median_multivariate_ = 67%; min_multivariate_ = 58%; max_multivariate_ = 75%). Thus, both shape proxies behave very similarly in accumulating adult shape features, and we propose that approx. 65% of the maximum adult body size is a reliable cut-off for sampling adult morphologies in turtle shells. The underlying species sample includes emydids, geoemydids, chelonioids, chelids, pelomedusoids, testudinids, and chelydroids, and thus represents a relatively broad phylogenetic spectrum of turtles.

Our ontogenetic shell shape curves show a phase of strong shell shape change that is generally followed by an asymptote or near-asymptotic levels at which shape converges onto an adult state (Figs. 2, 3). Our curves show varying degrees of plateauing of the Gompertz curves, and individual species can have different plateauing levels in CAC- or multivariate shape-based curves (Figs. 2, 3). Gompertz curves in CAC-graphs generally show better plateauing than multivariate shape-graphs. In some cases, the plateauing is likely affected by our data sampling, and larger sample sizes may capture the shape convergence levels of adult shape better than our preliminary data. However, the weakly plateauing Gompertz curves in some well-sampled species which include adults of both sexes near the absolute maximum size of the species (e.g., *Manouria emys*, *Trachemys scripta*) could also suggest that shape-growth does not closely follow a Gompertz function. Because of the varying plateauing levels across species, the lower 95% confidence intervals often provide unrealistic low size thresholds (mean = 50%, min = 3%, max = 82%; Supplementary Table 2; Supplementary Fig. 18) for adult shapes, which would imply that adult shapes are reached before sizes typical for sexual maturity. Thus, for our results and discussion, we largely focus on the thresholds implied by the 85% adult shape.

Although the clusters from the cluster analysis are based on the respective shape proxy and shell size, the ‘large’ cluster category does not uniformly fall together with the 85% shape thresholds or with the lower 5% confidence intervals on shape asymptotes. The 85% shape thresholds nearly ubiquitously fall within the ‘large’ cluster, indicating that the 85% cut-off is quite conservative within the context of multivariate shape and absolute shell size. On the contrary, the lower 5% confidence intervals on shape asymptotes usually fall within the ‘intermediate’ cluster, highlighting the uncertainty of the asymptotic approach to generating size thresholds in the current dataset.

### Sexual shape dimorphism

**Fig. 4:**
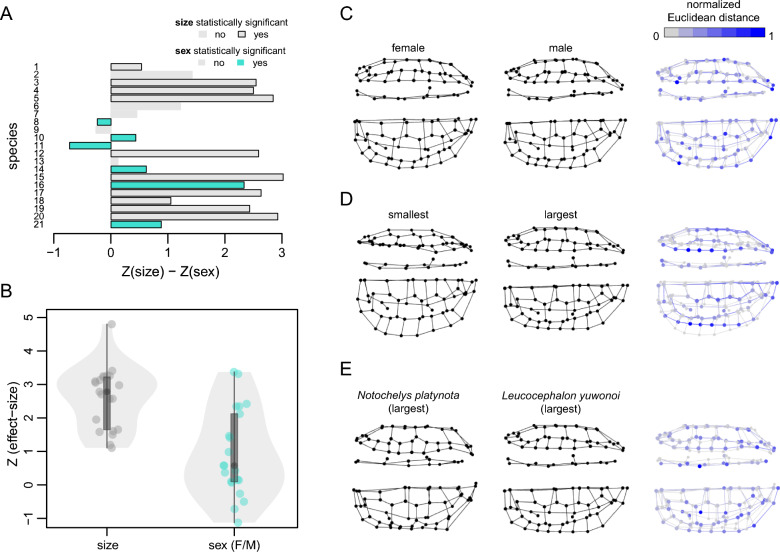
Comparisons of allometric and sexual dimorphism effects on shell shape for selected species. **A** Difference in effect-sizes (Z-scores) of ‘shell shape ~ size + sex’ regressions for 15 turtle species. Negative values indicate sex effects are larger than size effects. Numbers correspond to: 1 - *Astrochelys radiata*, 2 - *Centrochelys sulcata*, 3 - *Chelodina oblonga*, 4 - *Chelus fimbriata*, 5 - *Cuora flavomarginata*, 6 - *Dermatemys mawii*, 7-  *Emydura macquarii*, 8 - *Heosemys grandis*, 9 - *Heosemys annandalii*, 10 - *Kinosternon creaseri*, 11 - *Leucocephalon yuwonoi*, 12 - *Manouria emys*, 13 - *Mauremys sinensis*, 14 - *Mauremys rivulata*, 15 - *Orlitia borneensis*, 16 - *Pelomedusa subrufa*, 17 - *Phrynops hilarii*, 18 - *Podocnemis vogli*, 19 - *Stigmochelys pardalis*, 20 - *Trachemys ornata*, 21 - *Trachemys scripta*. **B** Violin plots showing the distributions of Z-scores of size and sex effects across species-specific regressions. **C** Landmark configurations of similar-sized female and male specimens (female FMNH 261568, SCL = 186 mm; male FLMNH 109835, SCL = 183 mm) in the species with the largest effect of sex in shell shape regressions (*Leucocephalon yuwonoi*). **D** Landmark configurations of the smallest (AMNH 145108, SCL = 67 mm) and largest (FLMNH 111310, SCL = 215 mm) specimens in the species with the largest effect of sex in shell shape regressions (*Leucocephalon yuwonoi*). **E** Landmark configurations of the largest specimen of the species with the largest sex effect on shell shape (*Leucocephalon yuwonoi*) and of the largest specimen of the most closely related species for which we had data (*Notochelys platynota*, FMNH 151017, SCL = 230 mm). Right column in (**C**–**E**) shows normalized Euclidean distances between landmarks of each configuration, whereby bluer points denote greater morphological change. For each species, shells are shown in left lateral (top rows) and dorsal views (bottom rows)

Allometric shape regressions with sex as a covariate show that sexual shape dimorphism is absent in many (N = 15) of the 21 tested turtles (Fig. 4A; Supplementary Table 3). In only six species, we received a significant effect of sex (Fig. 4A). In only two of these, the effect of sex is stronger than the effect of ontogeny, indicating that sex effect changes are less pronounced that ontogenetic shell shape changes (Fig. 4A). Overall, size has a median effect-size of 2.8, and its effect is thus ~ 4.5-fold the effect of sex, which has a median value of 0.6 (Fig. 4B). Shape comparisons of two same-sized female and male specimens of the turtle in which we detected the largest relative sex effects, the geoemydid *Leucocephalon yuwonoi*, show that shape differences are minor (Fig. 4C). These include a somewhat more elongated shell and more concave plastron in the male. However, these differences are minor when compared to ontogenetic shell shape differences in the species (Fig. 4D), which include strong shell elongation, and also when compared to interspecific differences with the closely related *Notochelys platynota* (Fig. 4E).

### Disparity analysis

**Fig. 5:**
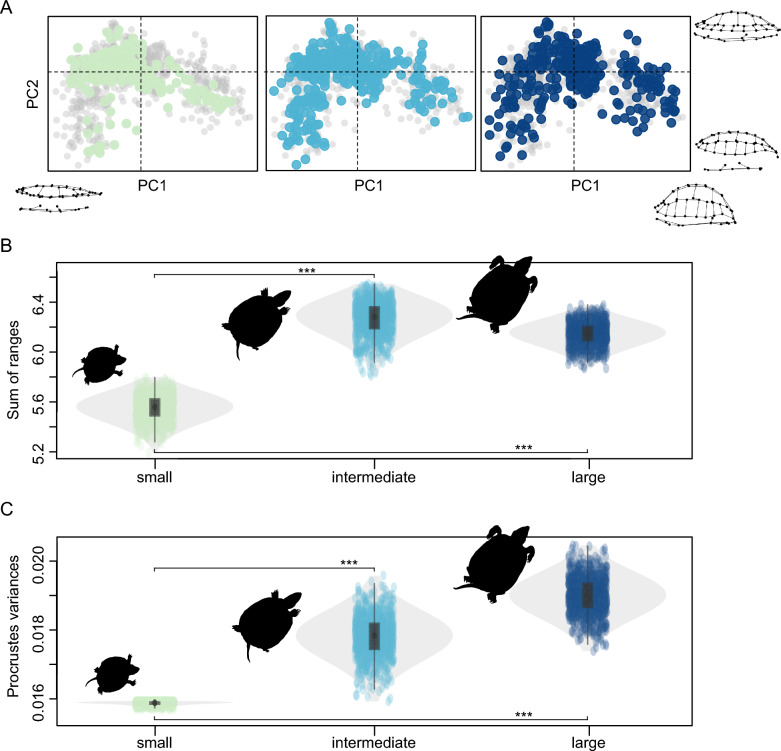
Morphological variation across ontogenetic stages in turtles. **A** First and second PCA axes illustrating main trends of turtle shell shape variation. Landmark configurations at the bottom indicate minimum (left) and maximum (right) PC1 values, whereas points on the right indicate minimum (bottom) and maximum (top) PC2 values. Grey points denote all specimens used in our analyses and different coloured points represent the different ontogenetic stages (‘small’, ‘intermediate’ and ‘large’). **B**, **C** Disparity ranges across ontogenetic stages in turtles using (**B**) sum of ranges (SoR) and (**C**) Procrustes variances. Asterisks (***) between ‘small’ and ‘intermediate’, and ‘small’ and ‘large’ groups indicate statistically non-overlapping confidence intervals of disparity values (P-values < 0.001; Table 2). Silhouettes are of different sized *Emys orbicularis* individuals in exhibition at the Natural History Museum of Fribourg (Switzerland)

Our disparity analysis results lend support to our initial hypothesis of an increased extent of shell shape differentiation throughout ontogeny (Fig. 5). Both of our disparity metrics used (sum of ranges and Procrustes variances) return the same patterns. ‘Small’ turtles exhibit significantly smaller morphological disparity in the shell than both ‘intermediate’ and ‘large’-sized turtles (Tables 1, 2). However, we find no statistical difference between the disparity ranges of ‘intermediate’ and ‘large’ turtle individuals (Table 2; Fig. 5B).

**Table 1:** Disparity analysis using sum of ranges (SoR)

Ontogenetic stages	N	Disparity	
		Sum of ranges	Procrustes variances
Small	195	5.95 [5.35–5.72]	0.01592 [0.0158–0.0160]
Intermediate	367	6.71 [5.99–6.45]	0.0180 [0.0167–0.0192]
Large	351	6.56 [5.97–6.33]	0.0190 [0.0178–0.0199]

Disparity results for sum of ranges and Procrustes variances, shown for each ontogenetic staged defined using the cluster analysis. Numbers outside brackets indicate observed disparity, whereas numbers in brackets indicate 95% confidence intervals. **N**, sample size

**Table 2:** Tests of overlapping confidence intervals

Group comparisons	Zou's statistic	p-value
Sum of ranges		
Small-intermediate	5.13	8.74 ∙10^–7^
Small-large	5.57	7.69 ∙10^–8^
Intermediate-large	0.60	1
Procrustes variances		
Small-intermediate	3.94	2.40 ∙10^–4^
Small-large	6.58	1.42 ∙10^–10^
Intermediate-large	1.38	0.50

Results from pairwise comparisons of overlapping confidence intervals (CI) of disparity values shown for both disparity metrics (sum of ranges and Procrustes variances). 95% CI values used in the tests are shown in Table 1. Zou’s statistic is based on CI overlap accounting for their standard errors (Zou, 2007 ). Values above 1.96 (at the 0.05 significance level) indicate non-overlapping CIs, whereas values below 1.96 point to no statistical difference between CI widths. P-values were adjusted using a Bonferroni correction

## Discussion

### Ontogenetic changes in turtle shell shape

Shell shape changes throughout growth in turtles, and we document common ontogenetic allometric trends across species. Small turtle individuals regardless of clade identity exhibit rounded carapaces (Fig. 1) and display a smaller range of morphological variation than adults (Fig. 5B, C). As turtles grow to intermediate and large body sizes, they tend to exhibit more elongate and narrower shells than juveniles (Fig. 1), which has also been noted by previous authors (e.g., Fish & Stayton, 2014; Pritchard, 2008). Nevertheless, the significantly expanded spread of the total morphological disparity of adults and already intermediately-sized turtles show that in addition, there are species-specific ontogenetic shape changes, such that adult turtles become more dissimilar to one another (Fig. 5).

In the vast majority of turtle species, shell shape changes slow down during late ontogeny, such that there is a species-specific adult morphology that individuals converge on. Although there is some variance across turtle species, adult shell shape morphologies are reached on average at about 65% of the maximum straight carapace length of a given species. Here, based on these preliminary assessments, we recommend this as a general size threshold for studies that want to subsample specimen-level datasets into adult-only datasets. These thresholds should be further scrutinized by data sampling for across the full growth spectrum of turtles, and we hope that this report of preliminary results will inspire more research on this topic.

Our inferred size threshold slightly exceeds size thresholds for sexual maturity, which in turtles are reached between 50 and 60% maximum straight carapace length (Avens & Snover, 2013; Hermanson & Evers, 2024; Kuchling, 1988; Mosimann & Bider, 1960; Pritchard & Trebbau, 1984; Webb, 1956). They also slightly exceed size thresholds for arrested growth rates for carapacial length in some sea turtle species, which happen at 46–60% (female vs. male) maximum shell size in *Chelonia mydas* (Supplementary Fig. 19) and at about 55–60% (female vs. male) maximum shell size in *Eretmochelys imbricata* (Bjorndal & Bolten, 1988; Kordikova, 2002). This indicates that our choice of defining adult shapes as 85% of the shape distance between juvenile shapes and record-size adult shapes is likely justified, as it makes sense that adult shapes are reached after sexual maturity or slowed growth rates are reached. Inferring adult shapes purely on asymptotic values from ontogenetic shell shape curves was largely unsuccessful, as Gompertz curves showed no uniform size threshold for convergence. We speculate that this is strongly influenced by our low sample size for many species.

### Disparity increase in adult shell shapes

There are multiple potential explanations for the increase in morphological differentiation during ontogeny, including distinct adaptations due to ontogenetic niche shifts, developmental constraints on juveniles, or pronounced adult sexual dimorphism. Rounder bodies perform better in turning, and may be an adaptation to avoid predation in juveniles (Myers et al., 2007; Stevens et al., 2018). Indeed, previous studies on extant cheloniids have shown that younger individuals tend to be more rounded, possibly as a defense against gape-limited predators (Salmon & Scholl, 2014; Salmon et al., 2015). Other studies have also highlighted the role of different incubation temperatures affecting hatchling turtle shell shapes (e.g., Mickelson & Downie, 2010; Ferreira-Júnior et al., 2011), although this has not been widely tested. Shell shape changes toward more oblong body shapes may reflect ontogenetic niche shifts (e.g., Pate & Salmon, 2017), as longer shapes facilitate maneuverability and a more stable swimming activity with reduced drag (Kamezaki & Matsui, 1997; Stevens et al., 2018). This may be a common trend in (crown-group) turtles, which are ancestrally aquatic such that terrestrial lineages represent secondary habitat shifts (Joyce & Gauthier,  2004; Sterli et al., 2018). Tortoises as an obligate terrestrial clade undergo the same patterns of decreased roundness in dorsal shell profiles due to relative length increase as aquatic turtles. However, tortoises also increase their roundness or doming in a coronal mid-shell profile during ontogeny by relative lateral flattening of the bridge peripherals, such that the ability for self-righting likely improves during ontogeny. Thus, we hypothesize that juveniles retain shells with suboptimal shapes for self-righting, possibly due to developmental constraints of their aquatic ancestry. The similarities in juveniles documented here may be more widely linked to common ancestry, whereby developmental constraints contribute to low juvenile disparity (Katz & Hale, 2016; Sherratt et al., 2017; e.g., by developmental canalization; Smith et al., 1985). Allometric differentiation then results in the appearance of defining shape features of lineages during postnatal development. A good example with previously documented evidence for this is the marked ontogenetic differences in the plastron of many hinged species (e.g., kinosternids and pelomedusids; Fig. 1D; Supplementary Figs. 5, 9), in which the hinges only arise during postnatal ontogeny, caused by tissue remodeling during growth (Cordero, 2023; Cordero et al., 2018).

### The effect of sex on shell shape is small

Our datasets consist of mixed sex data. This is potentially a major problem, because turtles can have pronounced sexual dimorphism. This is particularly well documented for body size (Berry & Shine, 1980; Pritchard, 2008; Halámková et al., 2013; Regis & Meik, 2017; TTWG, 2021), but sexual shape dimorphism is less well documented. For some turtles, shell shape dimorphism is reported to be small, such as in the tortoise *Manouria emys* (Stanford et al., 2015), or trionychids (Joyce, 2025). However, sexual dimorphism can be pronounced in some turtle species (Berry & Shine, 1980; Pritchard, 2008), with well known examples particularly in testudinoids (Das & Bhupathy, 2009; Kaddour et al., 2008; Lindeman, 1999; Mann et al., 2006; Schafer & Krekorian, 1983; Vega & Stayton, 2011; Willemsen & Hailey, 2003). Sexual size dimorphism does not necessarily translate into marked sexual shape dimorphism (e.g., Auliya et al., 2016; Broadley & Boycott, 2009; Figgener et al., 2022). Thus, sexual size and shape dimorphisms of turtle shells might not always be coupled (e.g., Adams et al., 2020; Cheng & Kuntner, 2015), so that strong sexual size dimorphism should not be seen as an indication that strong sexual shape dimorphism is expected.

Our data suggests that sexual shape dimorphism is small compared to sexual size dimorphism in turtle shells: Allometric regressions with sex as a covariate show that many species have no significant effect of sex on shape, and that the effects, when significant, are often smaller than ontogenetic effects. Small sex effects are measured in turtles with (e.g., *Trachemys scripta*) and without (e.g., *Kinosternon creaseri*, *Pelomedusa subrufa*) strong sexual size dimorphism, reinforcing the idea that sexual size and shape dimorphism are largely decoupled in turtles. The species with the strongest sex effect compared to size among our data, *Leucocephalon yuwonoi*, has only moderate sexual size dimorphism (around 15% size difference between the largest males and females; TTWG, 2021). Qualitative comparisons of this species’ sexual shape dimorphism with interspecific variation of even closely related species also suggest that the effect of sexual shape dimorphism is lower than the effect of taxonomy. Thus, whilst we think it is important to consider the ontogenetic stage of shape samples for turtles, our findings suggest that geometric morphometric studies focusing on inter-specific differences can be performed on mixed-sex datasets.

Although we measure only weak effects of sexual dimorphism in the context of our methods and data, effects of sexual dimorphism could contribute to higher adult shell shape disparities in respective species compared to species which lack pronounced sexual shell shape dimorphism. For example, although between-sex variation is indicated to be low due to the absence of a significant sex effect in allometric regressions of the testudinid *Centrochelys sulcata*, we noticed elevated variation within the adult male sample of the species. Whereas the multivariate shape values of all four male specimens are nearly identical, indicating they globally have very similar shapes in the context of the data, their CAC values show a larger spread, indicating that the size-related shape effects vary between these specimens. Inspection of the landmark data for the four largest available males for *Centrochelys sulcata* shows that a large proportion of the adult variability is caused by differences in the depth of the plastral concavity, which is one of the primary sexually dimorphic traits of many testudinids (Kaddour et al., 2008; McRae et al., 1981; Pritchard, 2008; Willemsen & Hailey, 2003). This suggests that adult variability linked to sexual shape dimorphism is not only due to intersex differences, but can even be related to within-sex variation of adults, likely because sexually dimorphic traits are more labile and plastic in adult populations (e.g., Hollander et al., 2006; Melnycky et al., 2013; Stewart et al., 2000). Some additional, known sexually variable aspects of shell shape, such as the anal notch roundness in pleurodires (e.g., Guerrero & Pérez-García, 2021; Medem, 1969; Páez et al., 2013; Pritchard & Trebbau, 1984) are currently not well captured in the landmark scheme, although the notch depth is recorded. These could possibly be implemented using semilandmark curves along bone margins (e.g., Dziomber et al., 2020; Hermanson et al., 2022).

Overall, assessment of sexual shape dimorphism requires better data. This may be of particular relevance for species with pronounced sexual size dimorphism (female- or male-biased), which can arise from different selection pressures (e.g., fecundity increase in females, combat success in males; Berry & Shine, 1980; Rhen & Lang, 1995), and thus result in faster growth rates leading to earlier acquisition of adult shell morphologies on either sex. Therefore, a more even sex sampling across the entire growth range of species is encouraged to further scrutinize our preliminary results.

## Conclusions

Turtles change shell shapes during ontogeny, but details of this process are not well documented across a broad phylogenetic spectrum of turtles. Using landmarked 3D shell shape measurements, we document ubiquitous shell shape changes in turtles and common pathways of change, which involve shell elongation from a rounded juvenile shape. Juvenile shapes are more similar across turtle clades than adult shapes, suggesting common developmental constraints that are overcome during lineage-specific post-natal ontogeny that brings forward shell shape changes between species.

We herein develop ontogenetic shell shape curves, which assess the development of shell shape changes across linear shell growth as an ontogenetic proxy. These curves indicate a convergence of shell shapes onto an adult morphology. Our data suggests a size threshold of 65% of the maximum per-species recorded straight carapace length can be generalized for turtles as the size at which 85% of the distance between juvenile and maximum-sized shell shape is reached. Sexual shell shape dimorphism is low across turtles even in the presence of pronounced sexual size dimorphism. Thus, mixed-sex datasets consisting of turtle shells that are at least 65% of the maximum recorded carapace length should be adequate for macroevolutionary assessments of shell shape.

Although the dataset we use is large, species-specific subsamples of the data can be quite small, such that our results are explicitly labelled as preliminary reports that provide guidance. Additional even and sex-specific sampling across the full growth range of turtle species will refine this work in the future.

## Supplementary Information

Supplementary Table 1.

Supplementary Table 2.

Supplementary Table 3.

Supplementary Text.

## Data Availability

The data generated and analysed in this study are available on GitHub (https://github.com/G-Hermanson/Turtle-shell-shape-ontogeny) and Zenodo (10.5281/zenodo.15690681).
