# Supplementary material for: PG-18: turtles reach adult shell shapes at about 65% maximum carapace length
Source: Swiss J Palaeontol. 2025 Aug 5;144(1):47. doi: 10.1186/s13358-025-00395-0 (PMC12325467; doi:10.1186/s13358-025-00395-0)
Supplement: Supplementary file 4 — Supplementary Text. [file 13358_2025_395_MOESM4_ESM.docx]

Supplementary Information to:

**PG-18: Turtles reach adult shell shapes at about 65% maximum carapace length**

Guilherme Hermanson, Serjoscha W. Evers

Correspondence to: guilhermehermanson@gmail.com; serjoscha.evers@googlemail.com

This PDF contains **Supplementary Figures 1–19** illustrating ***(i)*** the landmark configuration employed in this study, ***(ii)*** ontogenetic shape changes from smaller to larger individuals of species-specific datasets, and ***(iii)*** ontogenetic shell shape curves between the common allometric component (CAC; Mitteroecker et al., 2004) or multivariate shape (e.g., Adams & Nistri, 2010) of species-specific datasets and their straight carapace lengths (SCL, in mm).


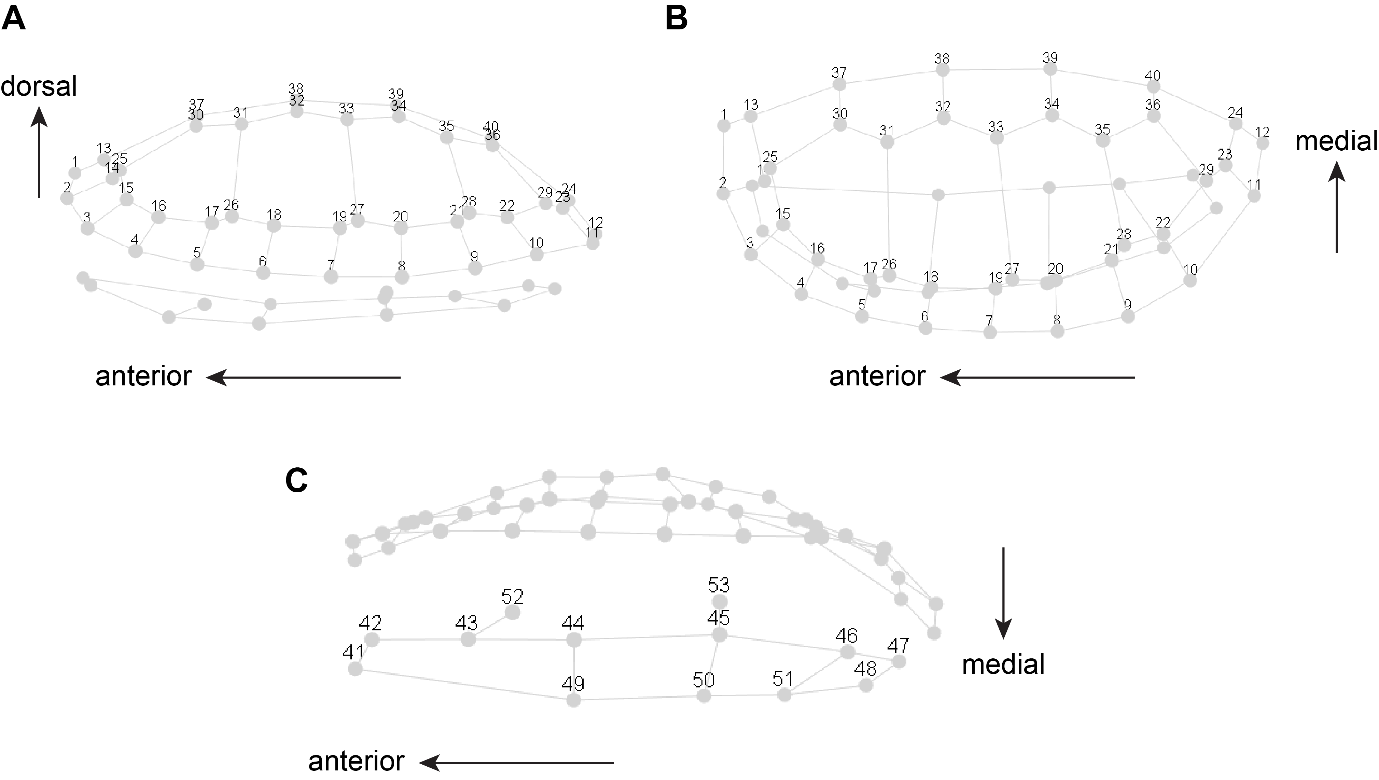


**Supplementary Figure 1. Landmark configuration.** 3D plots illustrating the landmark configuration used in this study, adopted from Stayton et al. (2018). The subpanels show the consensus shape output from the Generalized Procrustes analysis used to remove size, translation and orientation effects from the original, raw data. (A) and (B) highlight the position of landmarks in the carapace in left lateral and dorsal views, respectively, whereas (C) highlights landmarks used in the plastron, shown in an oblique ventral view. Note that the numbers differ from the sequence of landmarks shown in Stayton et al. (2018: fig. 1).


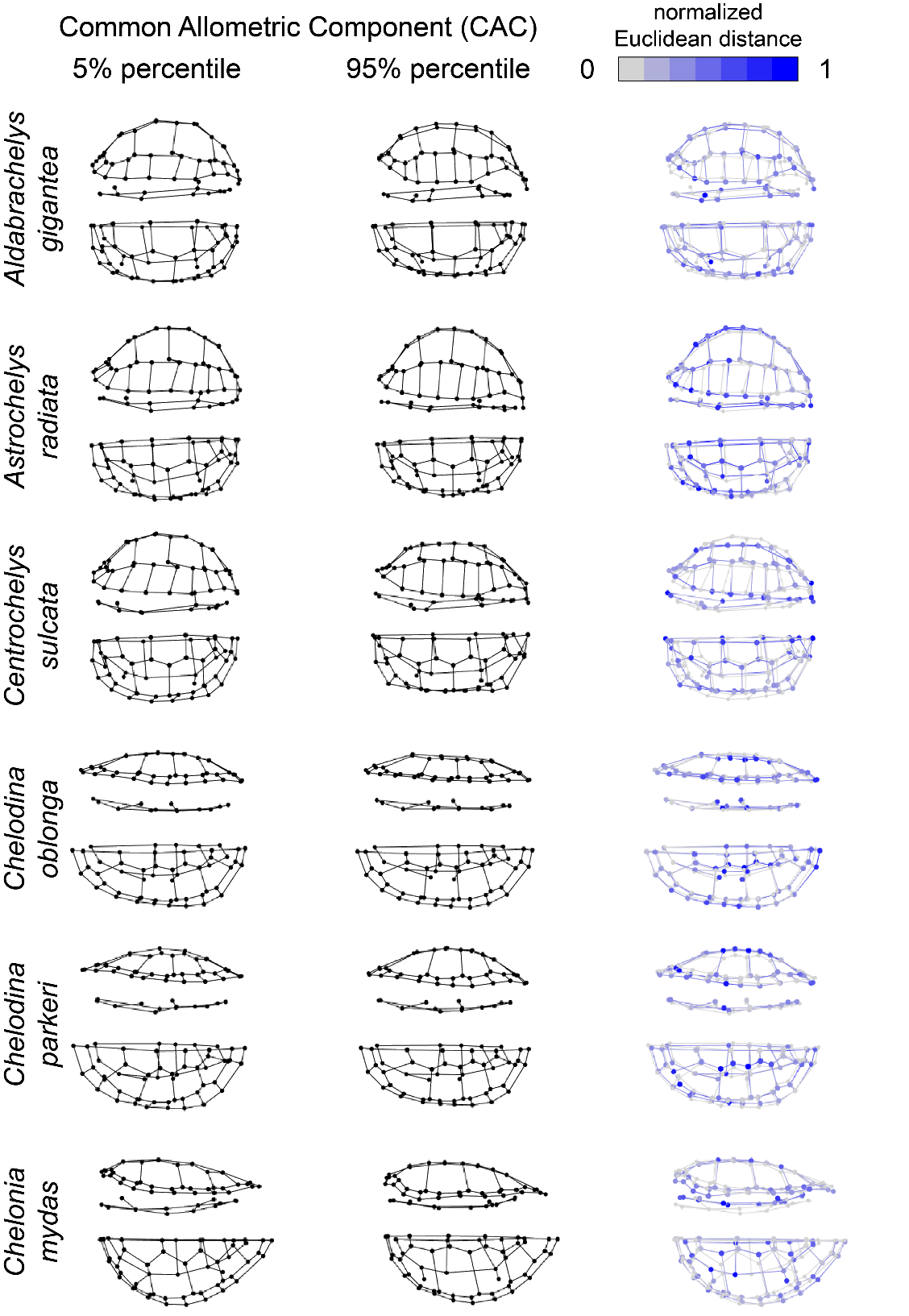


**Supplementary Figure 2. Ontogenetic shape changes in turtle shells across selected species with significant regressions and large adults sampled.** Landmark configurations shown for the 5% (left column) and 95% (middle column) percentiles of CAC values and normalized Euclidean distances between landmarks of each configuration (right column). Bluer points in the colour gradient denote greater morphological change. For each species, shells are shown in left lateral (top rows) and dorsal views (bottom rows).


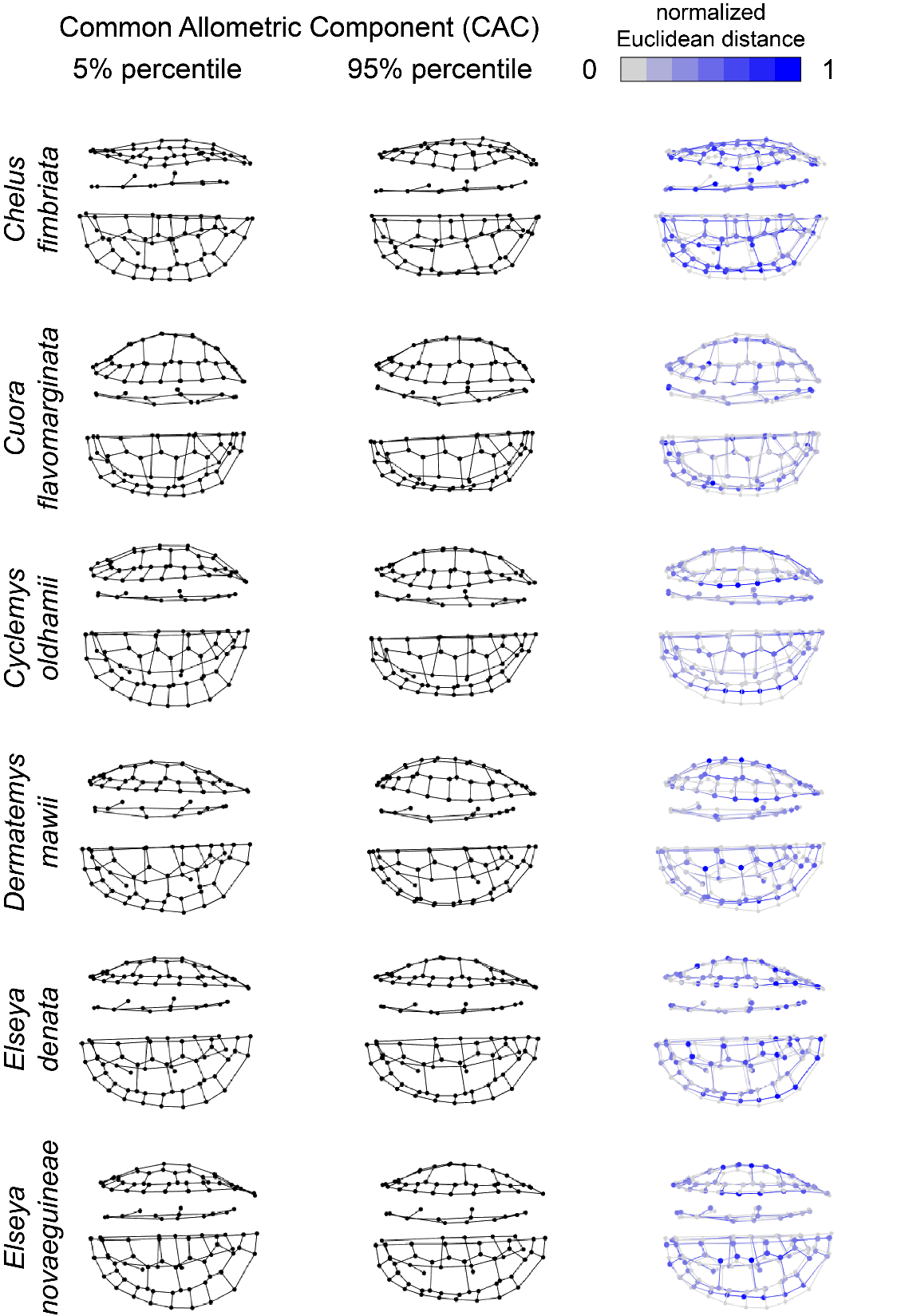


**Supplementary Figure 3. Ontogenetic shape changes in turtle shells across selected species with significant regressions and large adults sampled *(continued)*.** Landmark configurations shown for the 5% (left column) and 95% (middle column) percentiles of CAC values and normalized Euclidean distances between landmarks of each configuration (right column). Bluer points in the colour gradient denote greater morphological change. For each species, shells are shown in left lateral (top rows) and dorsal views (bottom rows).


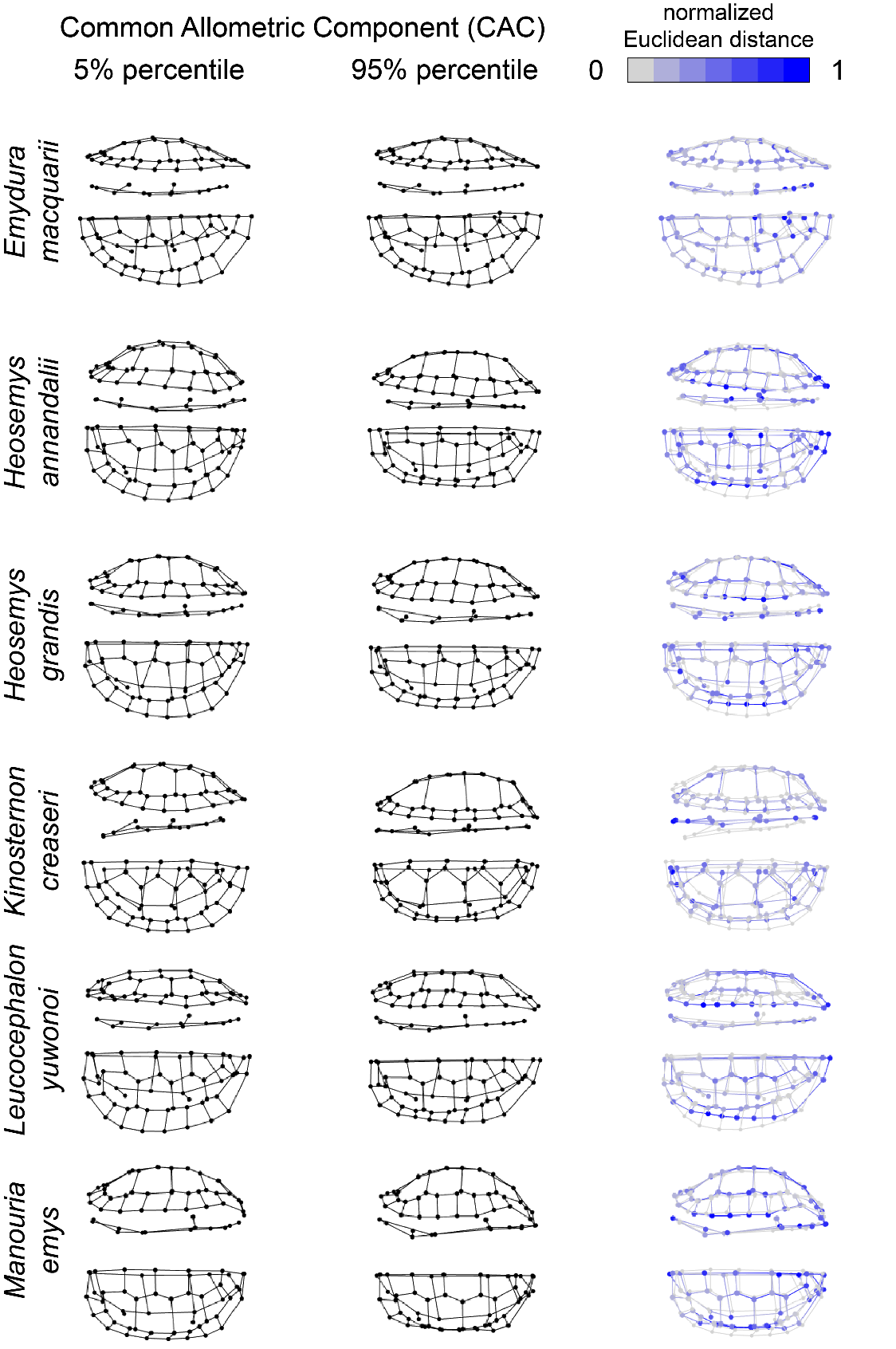


**Supplementary Figure 4. Ontogenetic shape changes in turtle shells across selected species with significant regressions and large adults sampled *(continued)*.** Landmark configurations shown for the 5% (left column) and 95% (middle column) percentiles of CAC values and normalized Euclidean distances between landmarks of each configuration (right column). Bluer points in the colour gradient denote greater morphological change. For each species, shells are shown in left lateral (top rows) and dorsal views (bottom rows).


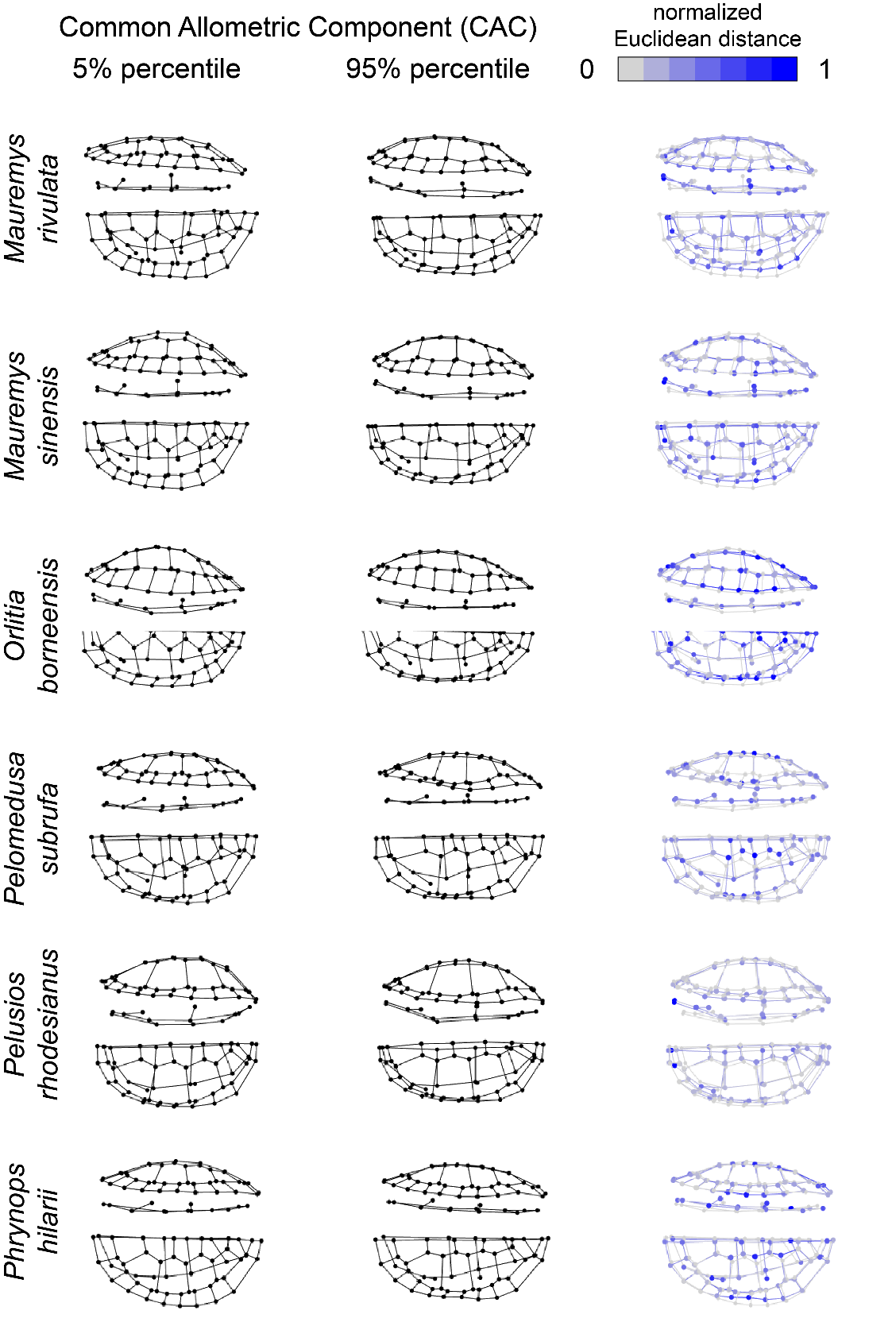


**Supplementary Figure 5. Ontogenetic shape changes in turtle shells across selected species with significant regressions and large adults sampled *(continued)*.** Landmark configurations shown for the 5% (left column) and 95% (middle column) percentiles of CAC values and normalized Euclidean distances between landmarks of each configuration (right column). Bluer points in the colour gradient denote greater morphological change. For each species, shells are shown in left lateral (top rows) and dorsal views (bottom rows).


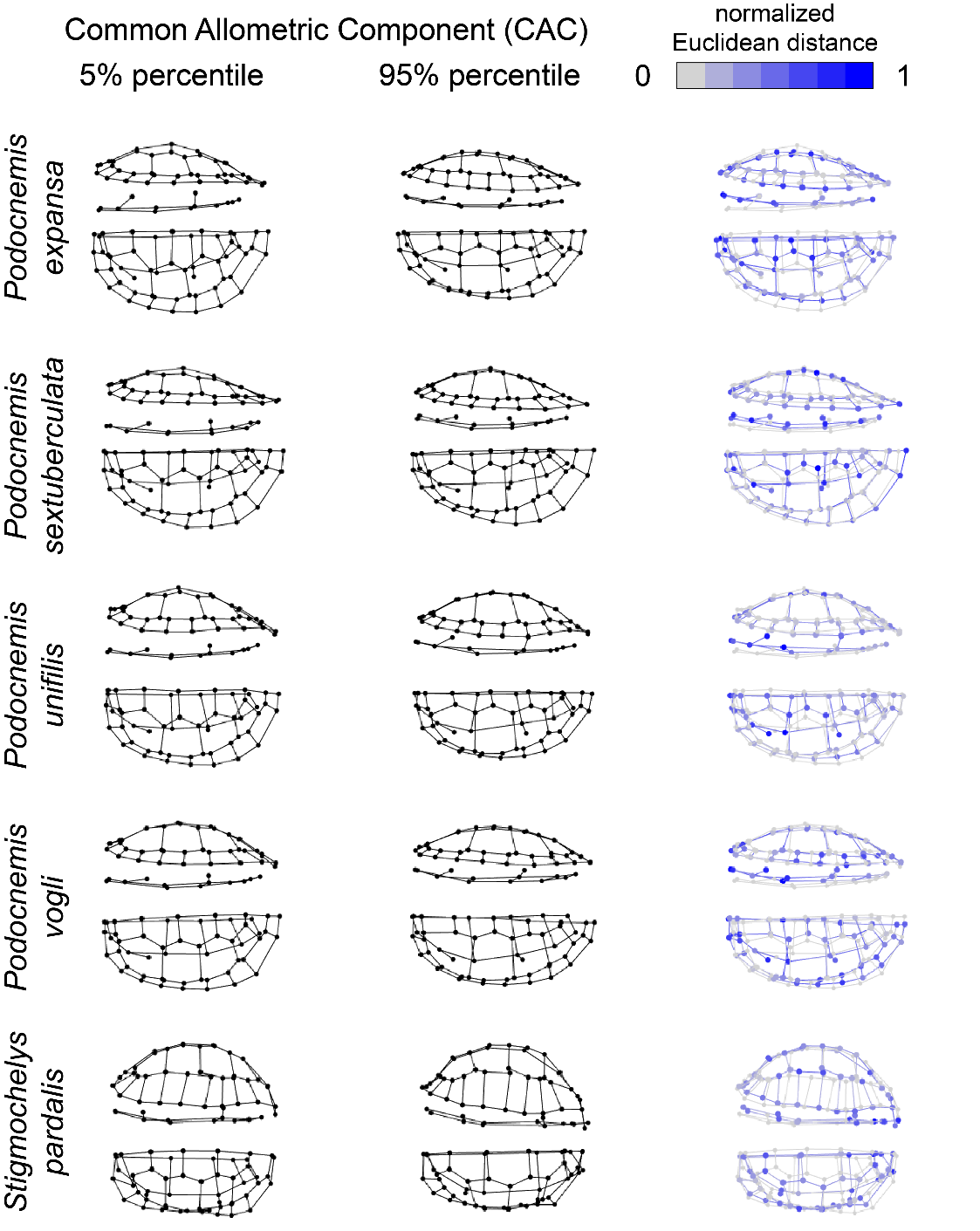


**Supplementary Figure 6. Ontogenetic shape changes in turtle shells across selected species with significant regressions and large adults sampled *(continued)*.** Landmark configurations shown for the 5% (left column) and 95% (middle column) percentiles of CAC values and normalized Euclidean distances between landmarks of each configuration (right column). Bluer points in the colour gradient denote greater morphological change. For each species, shells are shown in left lateral (top rows) and dorsal views (bottom rows).


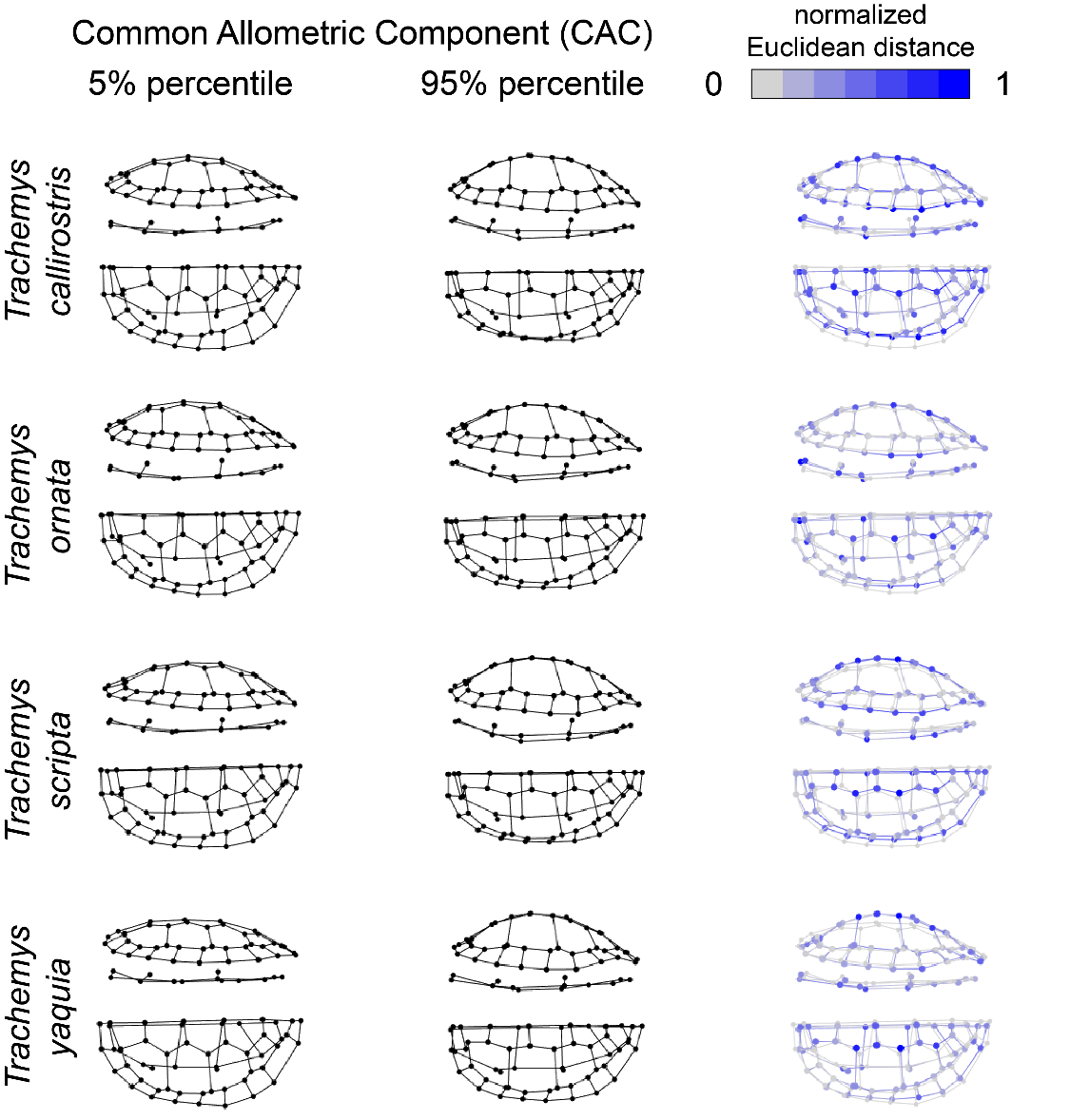


**Supplementary Figure 7. Ontogenetic shape changes in turtle shells across selected species with significant regressions and large adults sampled *(continued)*.** Landmark configurations shown for the 5% (left column) and 95% (middle column) percentiles of CAC values and normalized Euclidean distances between landmarks of each configuration (right column). Bluer points in the colour gradient denote greater morphological change. For each species, shells are shown in left lateral (top rows) and dorsal views (bottom rows).


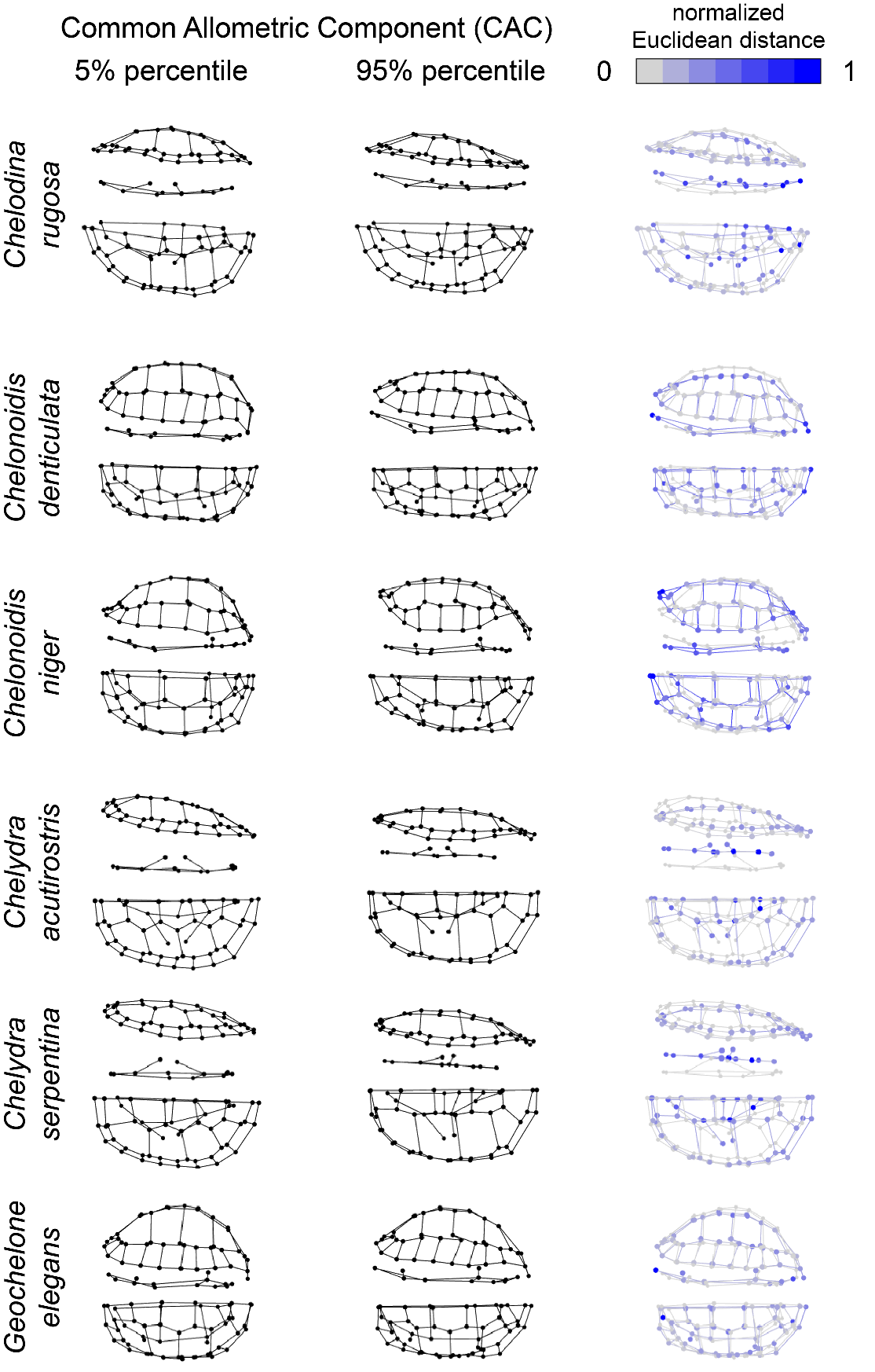


**Supplementary Figure 8. Ontogenetic shape changes in turtle shells across selected species with significant regressions but large adults unsampled.** Landmark configurations shown for the 5% (left column) and 95% (middle column) percentiles of CAC values and normalized Euclidean distances between landmarks of each configuration (right column). Bluer points in the colour gradient denote greater morphological change. For each species, shells are shown in left lateral (top rows) and dorsal views (bottom rows).


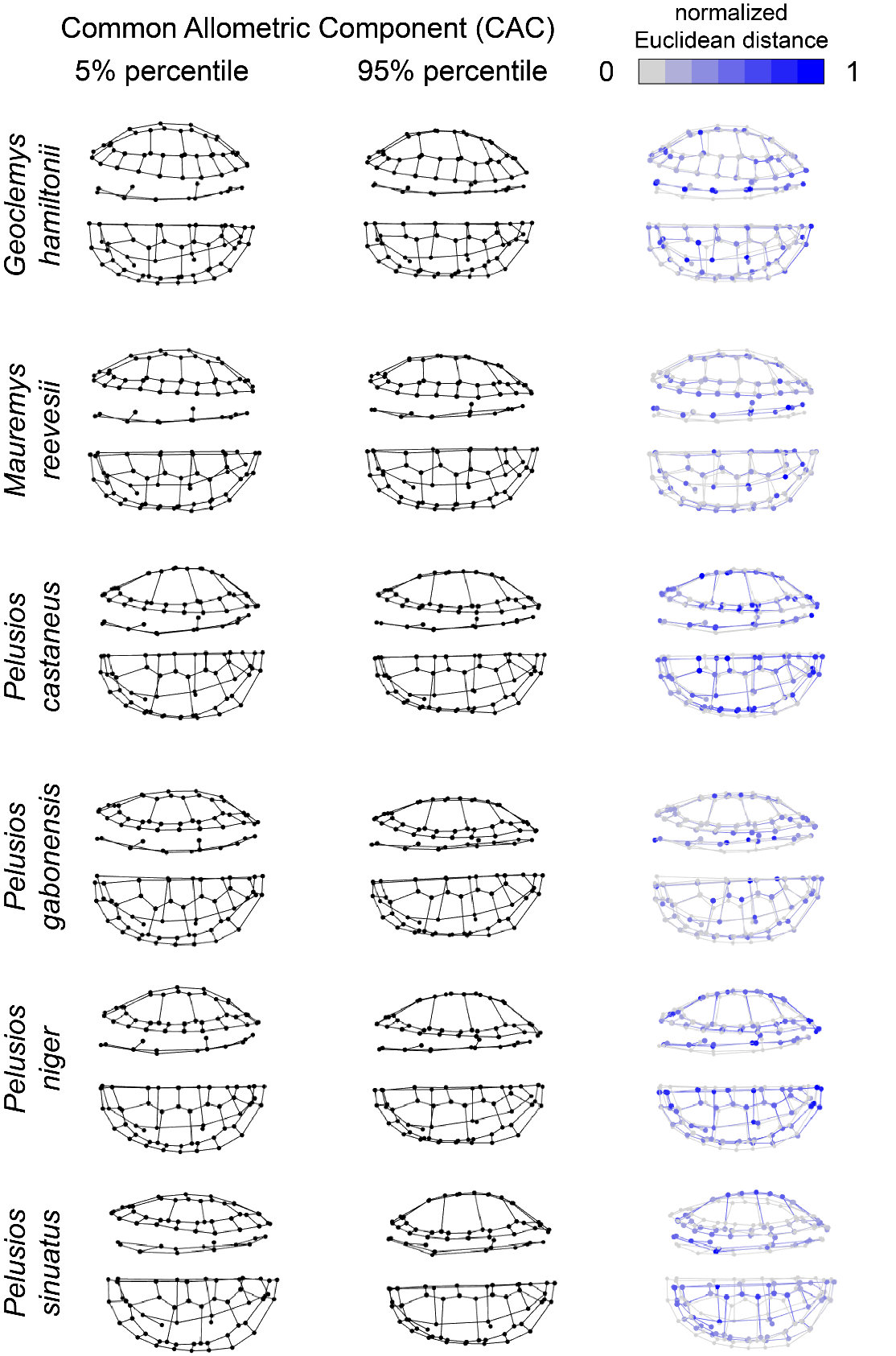


**Supplementary Figure 9. Ontogenetic shape changes in turtle shells across selected species with significant regressions but large adults unsampled *(continued)*.** Landmark configurations shown for the 5% (left column) and 95% (middle column) percentiles of CAC values and normalized Euclidean distances between landmarks of each configuration (right column). Bluer points in the colour gradient denote greater morphological change. For each species, shells are shown in left lateral (top rows) and dorsal views (bottom rows).


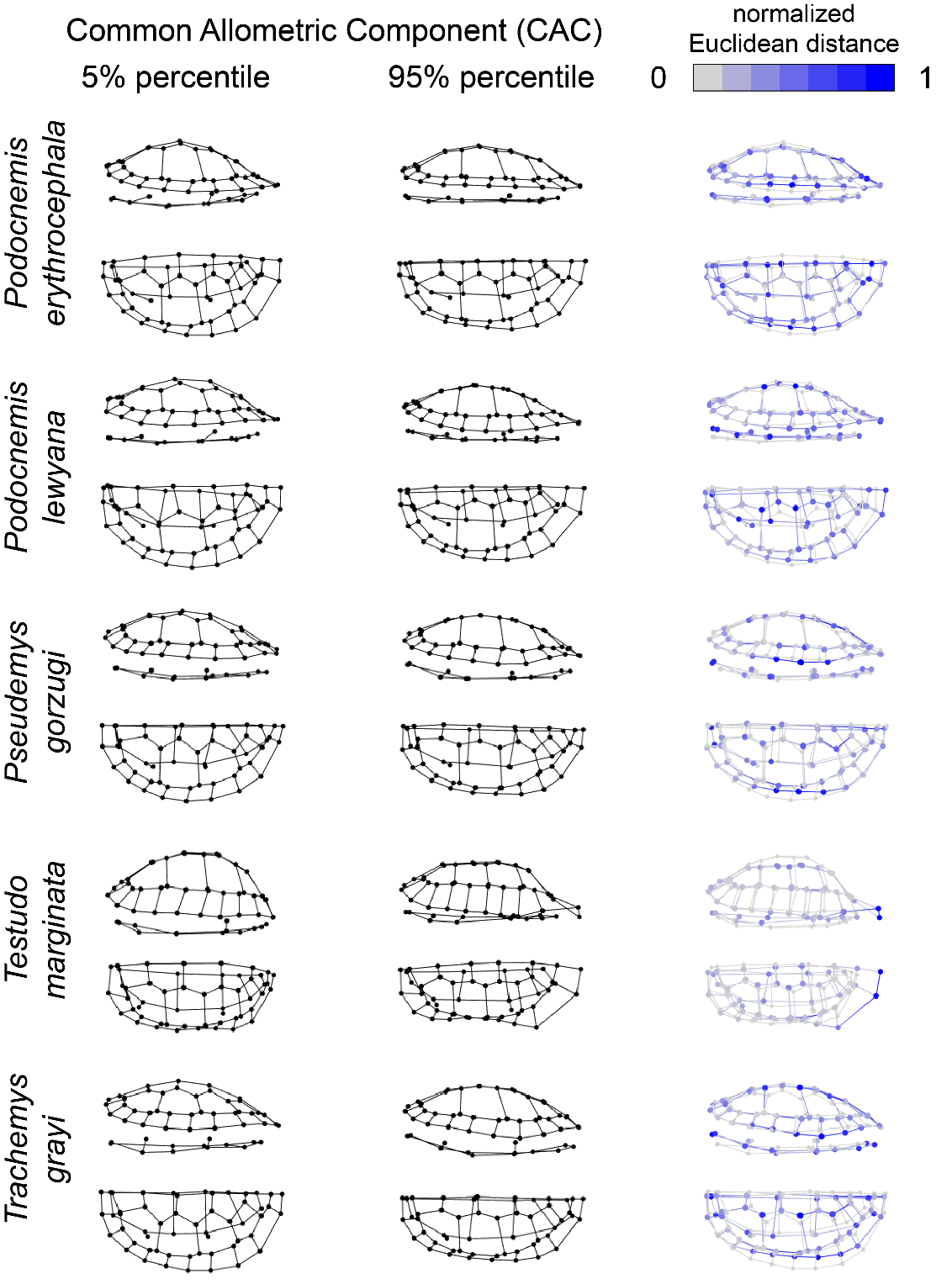


**Supplementary Figure 10. Ontogenetic shape changes in turtle shells across selected species with significant regressions but large adults unsampled *(continued)*.** Landmark configurations shown for the 5% (left column) and 95% (middle column) percentiles of CAC values and normalized Euclidean distances between landmarks of each configuration (right column). Bluer points in the colour gradient denote greater morphological change. For each species, shells are shown in left lateral (top rows) and dorsal views (bottom rows).


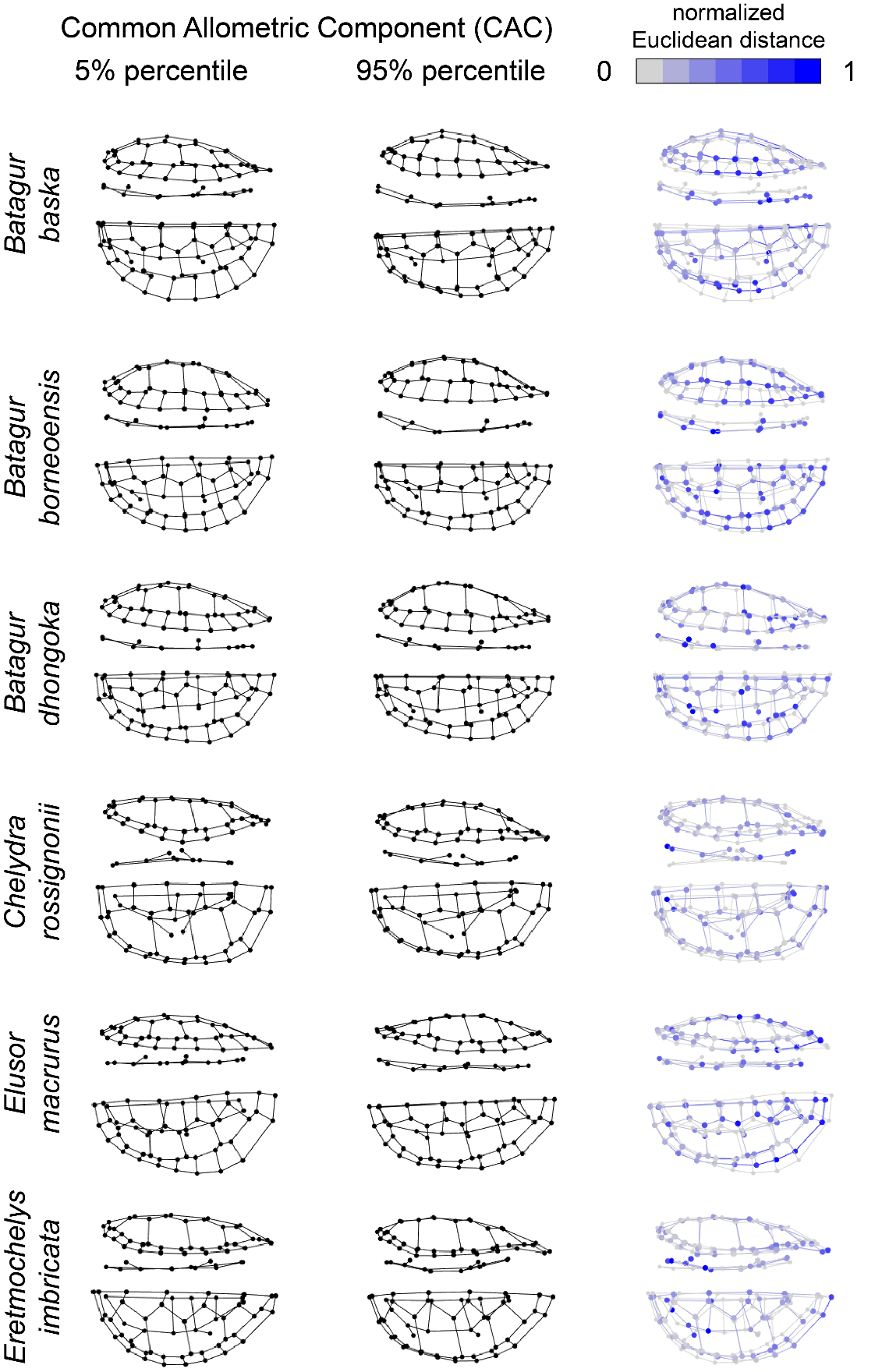


**Supplementary Figure 11. Ontogenetic shape changes in turtle shells across selected species with non-significant regressions.** Landmark configurations shown for the 5% (left column) and 95% (middle column) percentiles of CAC values and normalized Euclidean distances between landmarks of each configuration (right column). Bluer points in the colour gradient denote greater morphological change. For each species, shells are shown in left lateral (top rows) and dorsal views (bottom rows).


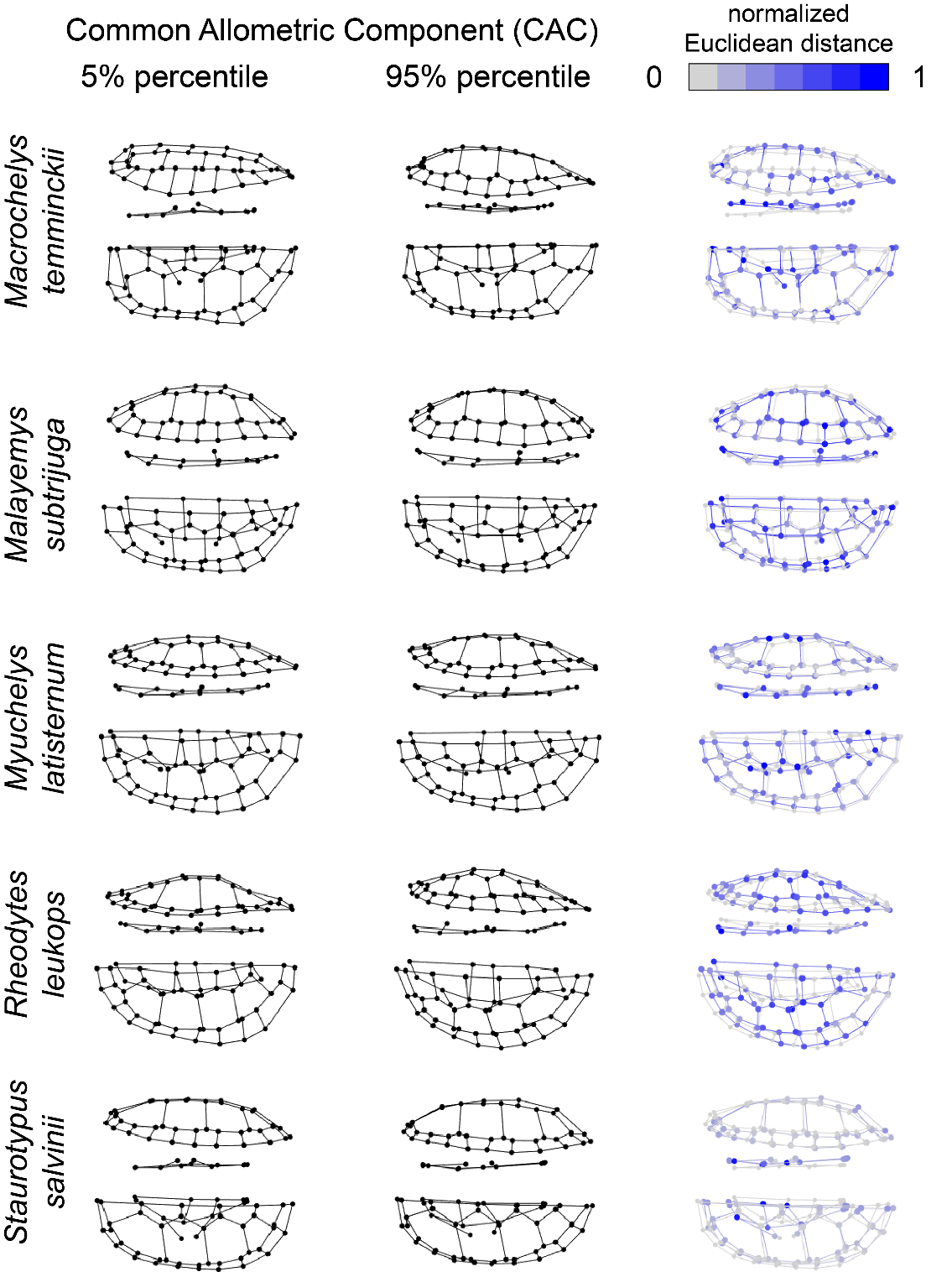


**Supplementary Figure 12. Ontogenetic shape changes in turtle shells across selected species with non-significant regressions *(continued)*.** Landmark configurations shown for the 5% (left column) and 95% (middle column) percentiles of CAC values and normalized Euclidean distances between landmarks of each configuration (right column). Bluer points in the colour gradient denote greater morphological change. For each species, shells are shown in left lateral (top rows) and dorsal views (bottom rows).


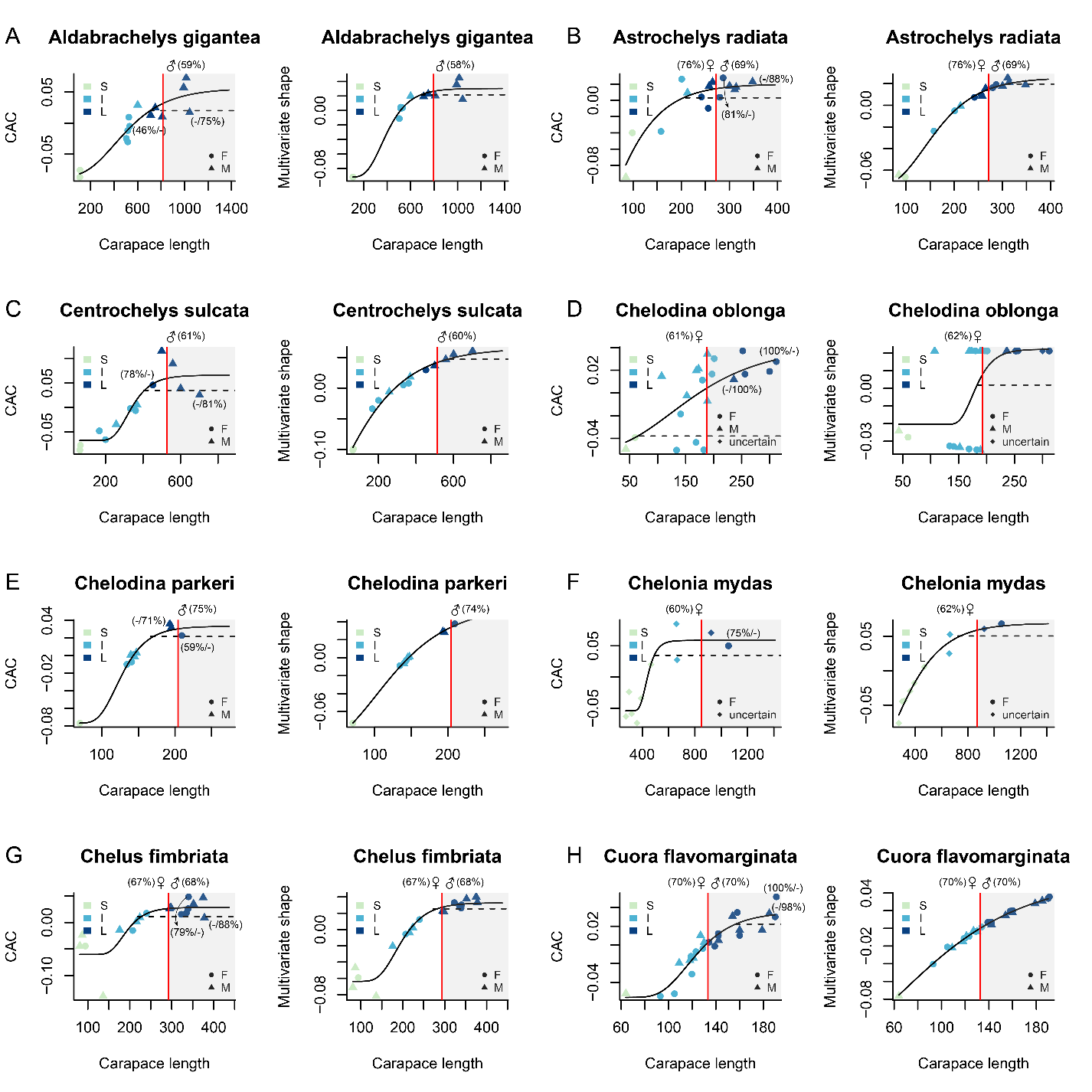


**Supplementary Figure 13. Ontogenetic shell shape curves for species with significant regressions and large adults sampled.** Bivariate plots of CAC (left) and multivariate shape (right) against straight carapace length. The size-range for adult CAC/multivariate shape, based on our 85%-adult shape threshold, is indicated by grey boxes. Red vertical lines show size threshold at which shape values reach 85% of the shape of maximum record-size specimens. Female/male (or both) percentages of maximum SCL recorded are indicated at the top of the line. Points are coloured according to their clusters (S = small, I = intermediate, L = large), and symbols represent sex. (X/Y%) values at largest datapoint indicates percentage of maximum female/male SCL recorded for the species. Solid lines are Gompertz curves fit across all data points. Dashed horizontal lines represent the lower 95% interval at which the asymptotic shape value is reached according to the Gompertz functions.


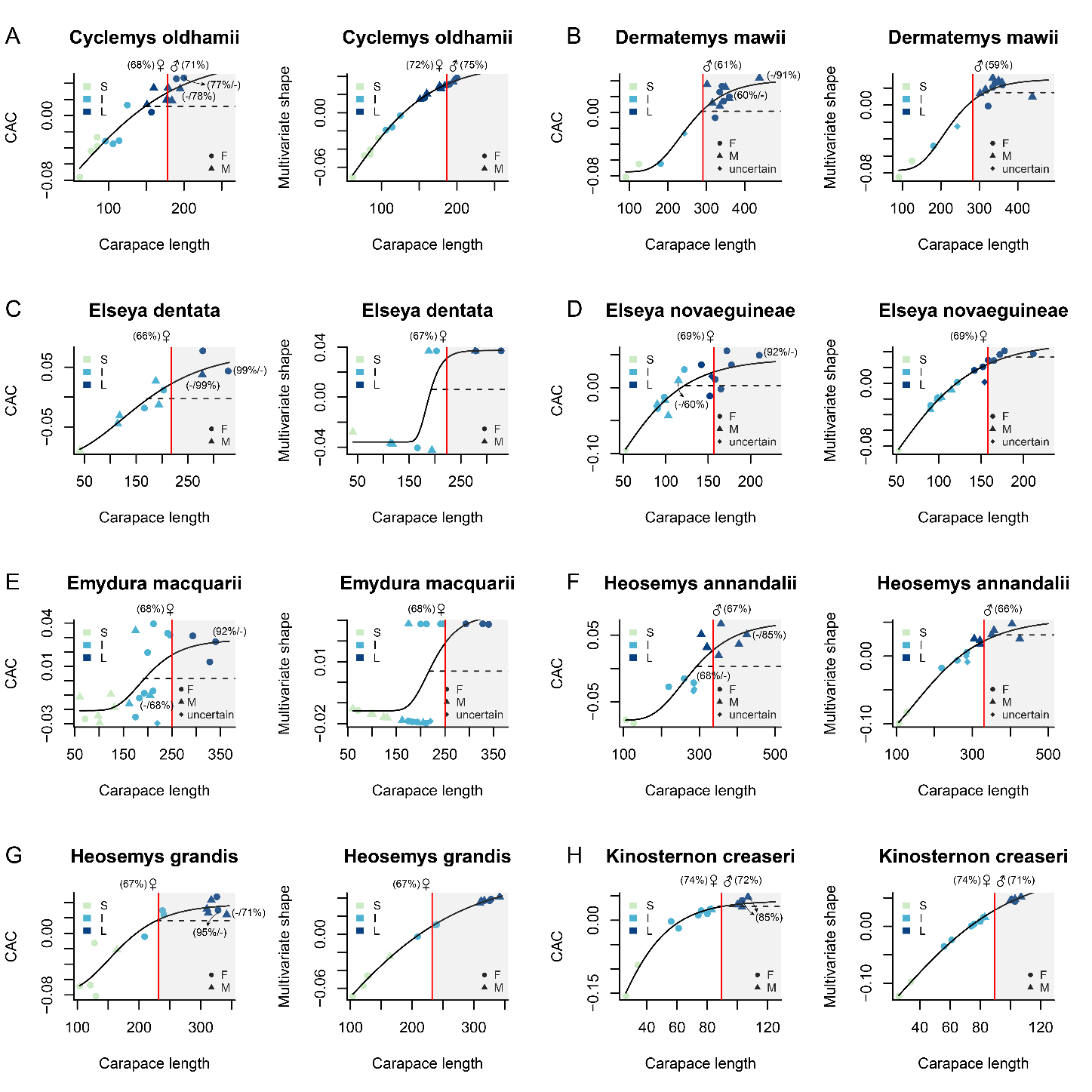


**Supplementary Figure 14. Ontogenetic shell shape curves for species with significant regressions and large adults sampled *(continued)*.** Bivariate plots of CAC (left) and multivariate shape (right) against straight carapace length. The size-range for adult CAC/multivariate shape, based on our 85%-adult shape threshold, is indicated by grey boxes. Red vertical lines show size threshold at which shape values reach 85% of the shape of maximum record-size specimens. Female/male (or both) percentages of maximum SCL recorded are indicated at the top of the line. Points are coloured according to their clusters (S = small, I = intermediate, L = large), and symbols represent sex. (X/Y%) values at largest datapoint indicates percentage of maximum female/male SCL recorded for the species. Solid lines are Gompertz curves fit across all data points. Dashed horizontal lines represent the lower 95% interval at which the asymptotic shape value is reached according to the Gompertz functions.


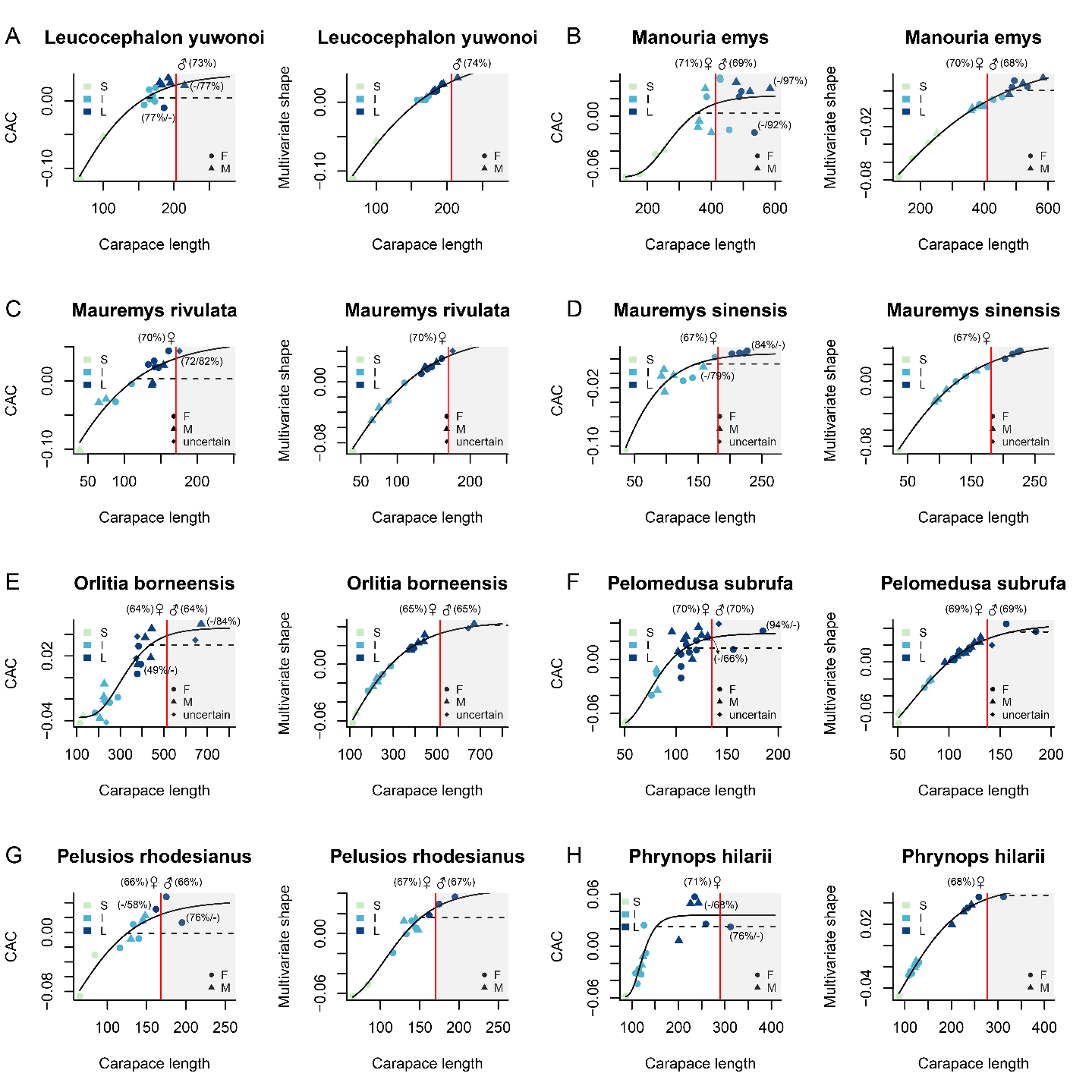


**Supplementary Figure 15. Ontogenetic shell shape curves for species with significant regressions and large adults sampled *(continued)*.** Bivariate plots of CAC (left) and multivariate shape (right) against straight carapace length. The size-range for adult CAC/multivariate shape, based on our 85%-adult shape threshold, is indicated by grey boxes. Red vertical lines show size threshold at which shape values reach 85% of the shape of maximum record-size specimens. Female/male (or both) percentages of maximum SCL recorded are indicated at the top of the line. Points are coloured according to their clusters (S = small, I = intermediate, L = large), and symbols represent sex. (X/Y%) values at largest datapoint indicates percentage of maximum female/male SCL recorded for the species. Solid lines are Gompertz curves fit across all data points. Dashed horizontal lines represent the lower 95% interval at which the asymptotic shape value is reached according to the Gompertz functions.


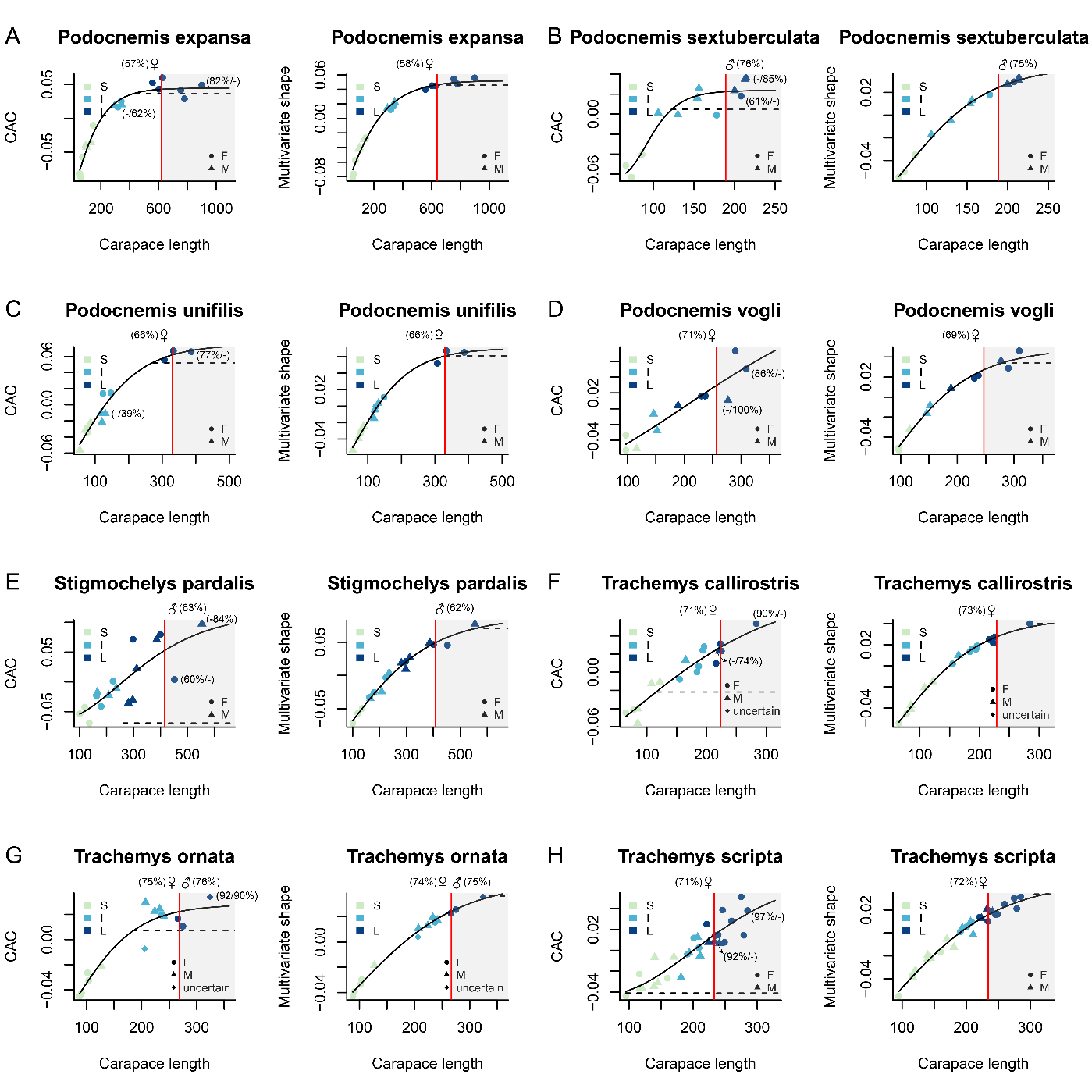


**Supplementary Figure 16. Ontogenetic shell shape curves for species with significant regressions and large adults sampled *(continued)*.** Bivariate plots of CAC (left) and multivariate shape (right) against straight carapace length. The size-range for adult CAC/multivariate shape, based on our 85%-adult shape threshold, is indicated by grey boxes. Red vertical lines show size threshold at which shape values reach 85% of the shape of maximum record-size specimens. Female/male (or both) percentages of maximum SCL recorded are indicated at the top of the line. Points are coloured according to their clusters (S = small, I = intermediate, L = large), and symbols represent sex. (X/Y%) values at largest datapoint indicates percentage of maximum female/male SCL recorded for the species. Solid lines are Gompertz curves fit across all data points. Dashed horizontal lines represent the lower 95% interval at which the asymptotic shape value is reached according to the Gompertz functions.


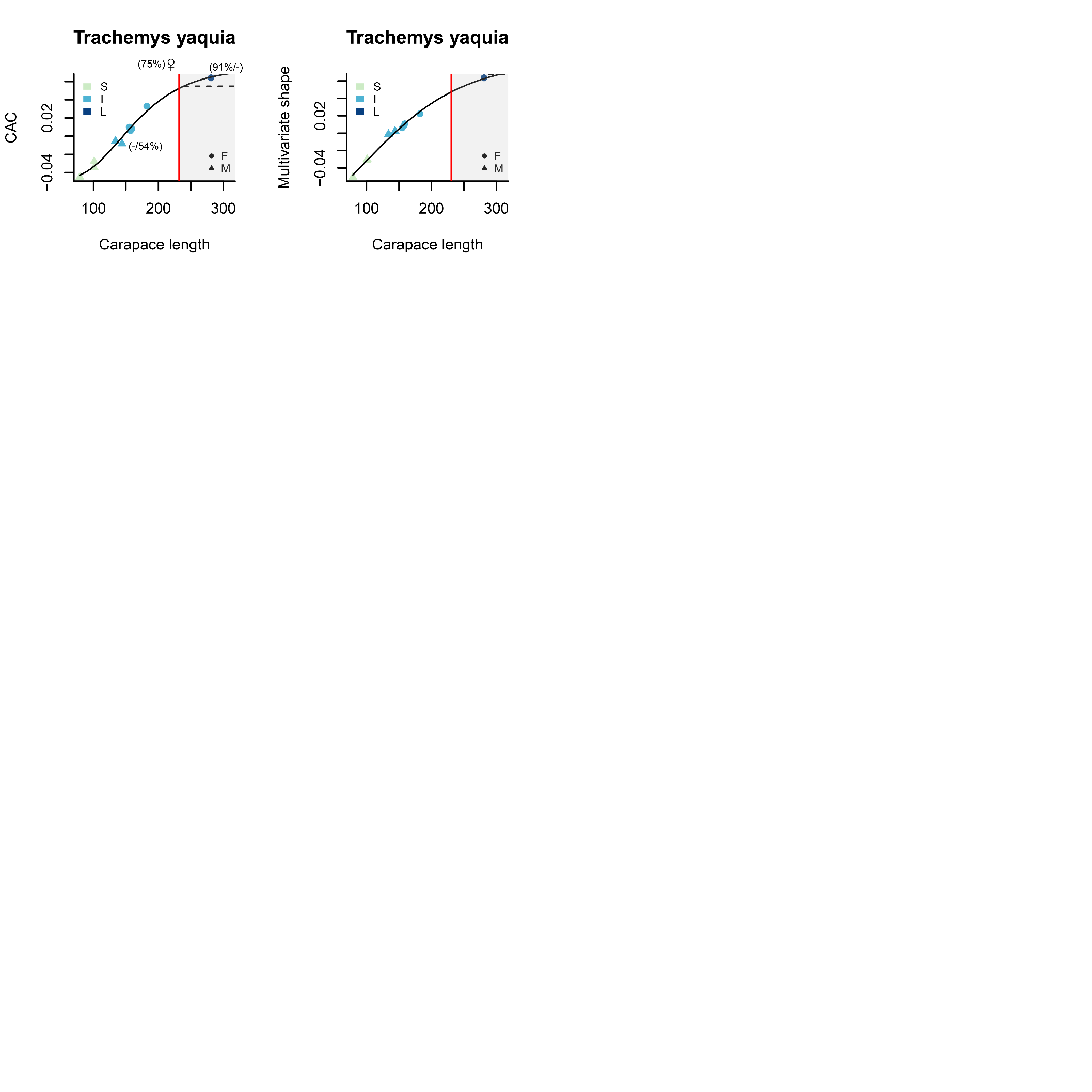


**Supplementary Figure 17. Ontogenetic shell shape curves for species with significant regressions and large adults sampled *(continued)*.** Bivariate plots of CAC (left) and multivariate shape (right) against straight carapace length. The size-range for adult CAC/multivariate shape, based on our 85%-adult shape threshold, is indicated by grey boxes. Red vertical lines show size threshold at which shape values reach 85% of the shape of maximum record-size specimens. Female/male (or both) percentages of maximum SCL recorded are indicated at the top of the line. Points are coloured according to their clusters (S = small, I = intermediate, L = large), and symbols represent sex. (X/Y%) values at largest datapoint indicates percentage of maximum female/male SCL recorded for the species. Solid lines are Gompertz curves fit across all data points. Dashed horizontal lines represent the lower 95% interval at which the asymptotic shape value is reached according to the Gompertz functions.


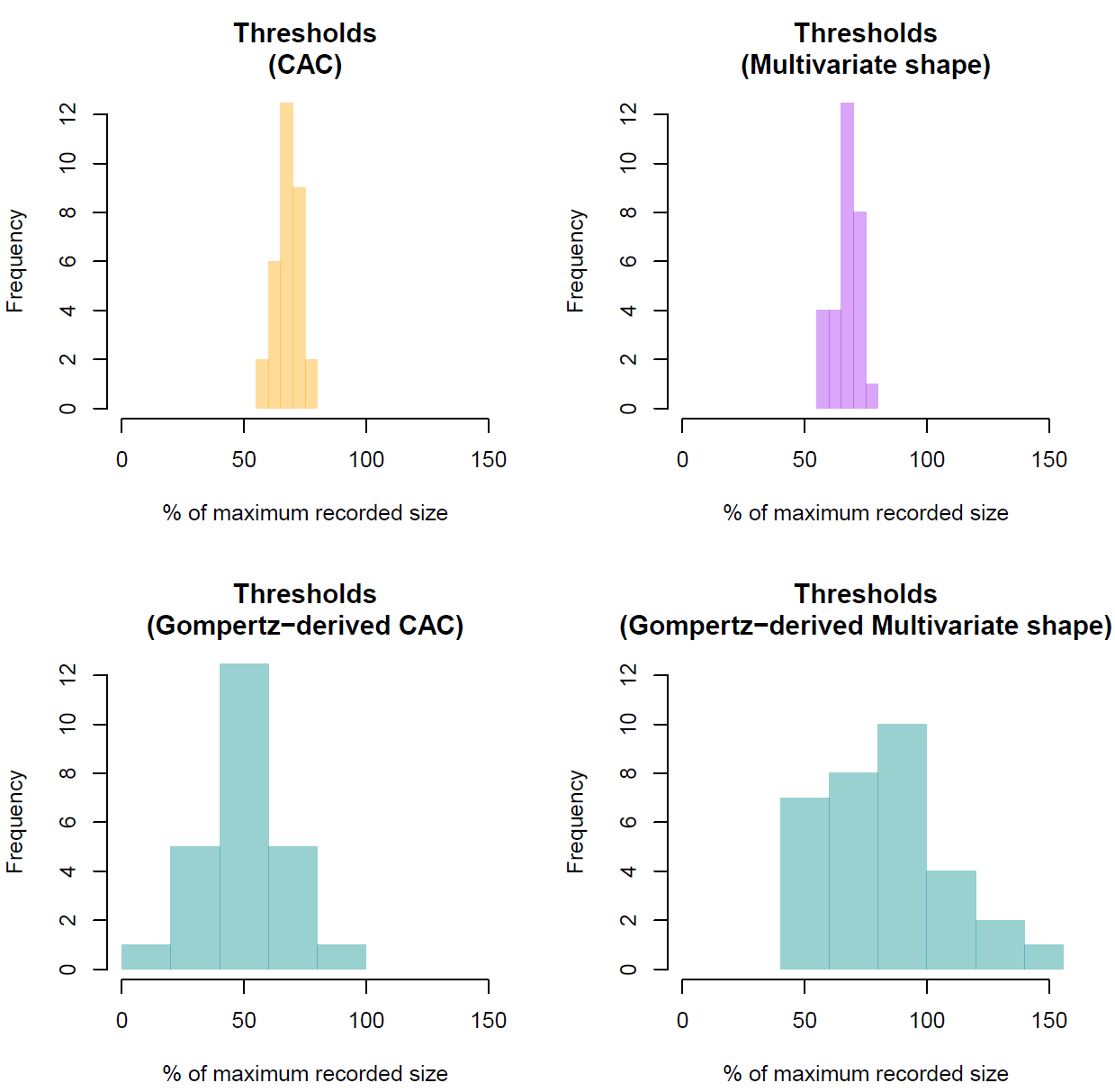


**Supplementary Figure 18. Size threshold values across different analytical pipelines.** Size thresholds (in %) inferred for ‘adult’ shell shapes according to CAC (top left), multivariate shape (top right) and Gompertz-derived methods (bottom row). Note large uncertainty of Gompertz-based functions compared to CAC or multivariate shape predictions of size thresholds. See Supplementary Table 3 for species-specific values of size thresholds.

**
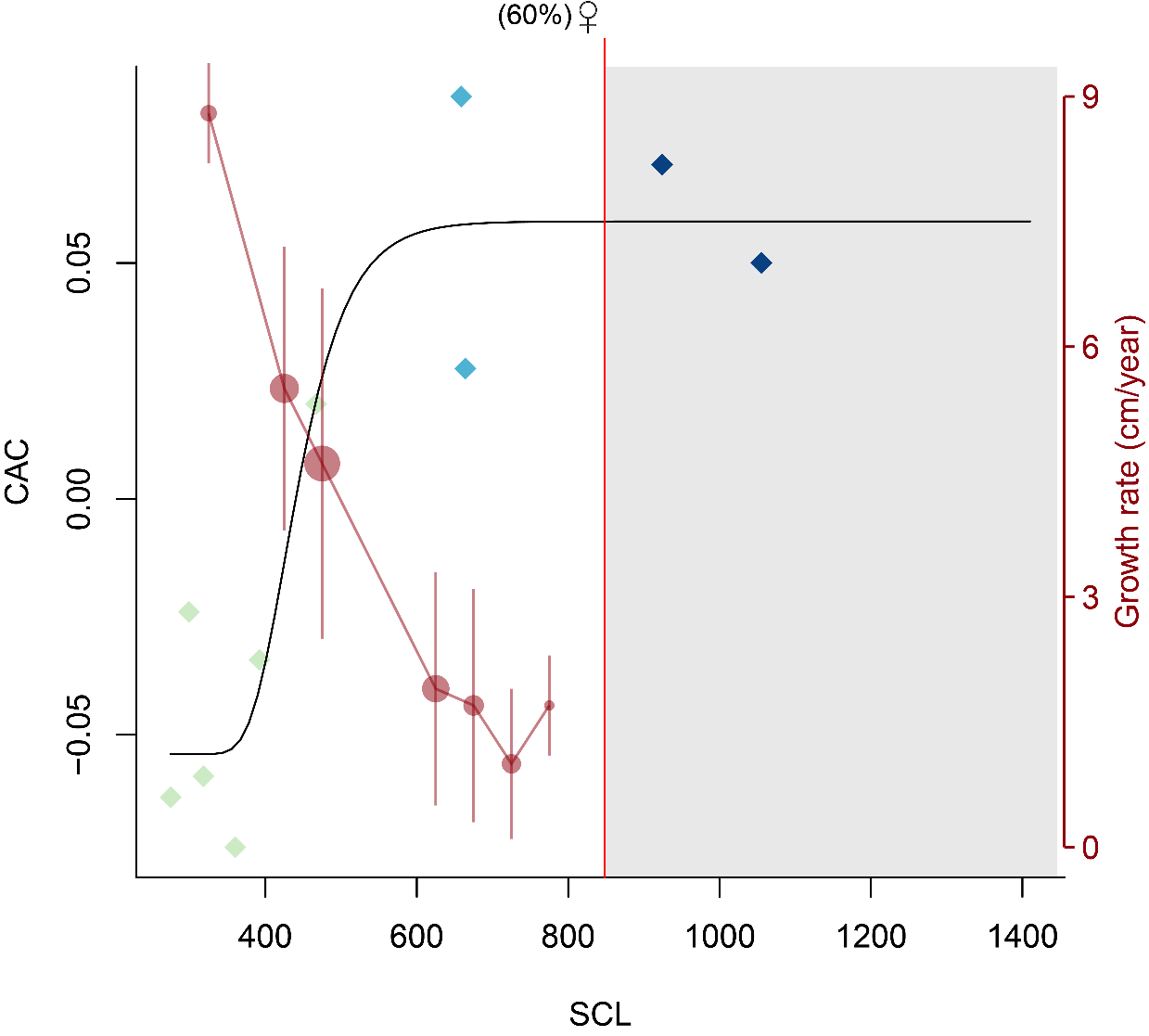
**

**Supplementary Figure 19.** **Ontogenetic shell shape curve of *Chelonia mydas*.** Combined plot of CAC values (left *y* axis) and carapace growth rates (right *y* axis) against straight carapace length of *Chelonia mydas*. The size-range for adult CACCAC convergence shape, based on our 85%-adult shape threshold, is indicated by grey boxes. The red vertical line indicates the size threshold at which CAC values reach 85% of the shape of record-sized specimens, with corresponding female percentages of maximum SCL indicated at the top of the line. Datapoints are colour-coded according to their cluster assignments (‘small’, ‘intermediate’ or ‘large’; see above). Growth rate data from Bjorndal and Bolten (1988) and Kordikova (2002). Size of points in the growth rate curve correspond to the sample size for a determined size class (minimum = 2, maximum = 24). Vertical lines across the points represent the standard deviation for each size class sample.

**References**

Adams, D.C., & Nistri, A. (2010). Ontogenetic convergence and evolution of foot morphology in European cave salamanders (Family: Plethodontidae). BMC Evolutionary Biology, 10, 1–10.

Bjorndal, K.A., & Bolten, A.B. (1988). Growth rates of immature green turtles, *Chelonia mydas*, on feeding grounds in the southern Bahamas. Copeia, 3, 555–564.

Kordikova, E.G. (2002). Heterochrony in the evolution of the shell of Chelonia. Part 1: terminology, Cheloniidae, Dermochelyidae, Trionychidae, Cyclanorbidae and Carettochelyidae. Neues Jahrbuch für Geologie und Paläontologie-Abhandlungen, 226(3), 343–413.

Mitteroecker, P., Gunz, P., Bernhard, M., Schaefer, K., & Bookstein, F.L. (2004). Comparison of cranial ontogenetic trajectories among great apes and humans. Journal of Human Evolution, 46(6), 679–698.

Stayton, C.T., O’Connor, L.F., & Nisivoccia, N.M. (2018). The influence of multiple functional demands on morphological diversification: a test on turtle shells. Evolution, 72(9), 1933–1949.

Turtle Taxonomy Working Group, Rhodin, A.G.J., Iverson, J.B., Bour, R., Fritz, U., Georges, A., Shaffer, H.B., & van Dijk, P.P. (2021). Turtles of the world, 9th edition: Annotated checklist of taxonomy, synonymy, distribution, and conservation status. Chelonian Research Monographs, 8, 1–472.
